# Selective targeting of α7 nicotinic acetylcholine receptor by synthetic peptide mimicking loop I of human SLURP-1 provides efficient and prolonged therapy of epidermoid carcinoma *in vivo*

**DOI:** 10.3389/fcell.2023.1256716

**Published:** 2023-10-03

**Authors:** O. V. Shlepova, M. A. Shulepko, V. O. Shipunova, M. L. Bychkov, I. D. Kukushkin, I. A. Chulina, V. N. Azev, E. I. Shramova, V. A. Kazakov, A. M. Ismailova, Y. A. Palikova, V. A. Palikov, E. A. Kalabina, E. A. Shaykhutdinova, G. A. Slashcheva, E. A. Tukhovskaya, I. A. Dyachenko, A. N. Murashev, S. M. Deyev, M. P. Kirpichnikov, Z. O. Shenkarev, E. N. Lyukmanova

**Affiliations:** ^1^ NTI Center, Shemyakin-Ovchinnikov Institute of Bioorganic Chemistry, Russian Academy of Sciences, Moscow, Russia; ^2^ Moscow Institute of Physics and Technology, National Research University, Dolgoprudny, Russia; ^3^ Faculty of Biology, Shenzhen MSU-BIT University, Shenzhen, China; ^4^ Immunology Department, Shemyakin-Ovchinnikov Institute of Bioorganic Chemistry, Russian Academy of Sciences, Moscow, Russia; ^5^ Bioengineering Department, Shemyakin-Ovchinnikov Institute of Bioorganic Chemistry, Russian Academy of Sciences, Moscow, Russia; ^6^ Branch of Shemyakin-Ovchinnikov Institute of Bioorganic Chemistry RAS, Pushchino, Russia; ^7^ Biomarker Research Laboratory, Institute of Fundamental Medicine and Biology, Kazan Federal University, Kazan, Russia; ^8^ Sechenov First Moscow State Medical University (Sechenov University), Moscow, Russia; ^9^ Interdisciplinary Scientific and Educational School of Moscow University Molecular Technologies of the Living Systems and Synthetic Biology, Faculty of Biology, Lomonosov Moscow State University, Leninskie Gory, Moscow, Russia; ^10^ Structural Biology Department, Shemyakin-Ovchinnikov Institute of Bioorganic Chemistry, Russian Academy of Sciences, Moscow, Russia

**Keywords:** cancer, α7-nAChR, Ly6/uPAR, SLURP-1, lynx1, xenograft, A431

## Abstract

α7-Type nicotinic acetylcholine receptor (α7-nAChR) promotes the growth and metastasis of solid tumors. Secreted Ly6/uPAR-Related Protein 1 (SLURP-1) is a specific negative modulator of α7-nAChR produced by epithelial cells. Here, we investigated mechanisms of antiproliferative activity of recombinant SLURP-1 in epidermoid carcinoma A431 cells and activity of SLURP-1 and synthetic 21 a.a. peptide mimicking its loop I (Oncotag) in a xenograft mice model of epidermoid carcinoma. SLURP-1 inhibited the mitogenic pathways and transcription factors in A431 cells, and its antiproliferative activity depended on α7-nAChR. Intravenous treatment of mice with SLURP-1 or Oncotag for 10 days suppressed the tumor growth and metastasis and induced sustained changes in gene and microRNA expression in the tumors. Both SLURP-1 and Oncotag demonstrated no acute toxicity. Surprisingly, Oncotag led to a longer suppression of pro-oncogenic signaling and downregulated expression of pro-oncogenic miR-221 and upregulated expression of KLF4 protein responsible for control of cell differentiation. Affinity purification revealed SLURP-1 interactions with both α7-nAChR and EGFR and selective Oncotag interaction with α7-nAChR. Thus, the selective inhibition of α7-nAChRs by drugs based on Oncotag may be a promising strategy for cancer therapy.

## 1 Introduction

The α7-type nicotinic acetylcholine receptor (α7-nAChR) is a homopentameric ligand-gated ion channel permeable to Ca^2+^ (Albuquerque et al., 2009). α7-nAChR is widely expressed in the brain (Kulbatskii et al., 2018) and also in non-neuronal tissues (Kurzen et al., 2004; Wessler and Kirkpatrick, 2009; Zoli et al., 2018), being involved in regulation of differentiation and apoptosis of epithelial cells (Grando, 2006), cytokine release by macrophages (Pavlov and Tracey, 2005), antibody production by B-cells (Kawashima et al., 2007), and T-cell differentiation (Mashimo et al., 2019).

Pro-oncogenic role of α7-nAChR is well documented (Grando, 2014; Schaal and Chellappan, 2014; Wang and Hu, 2018; Hollenhorst and Krasteva-Christ, 2021). Activation of α7-nAChRs promotes growth and metastasis of pancreatic ductal adenocarcinomas (Schaal et al., 2015), proliferation and invasion of breast (Dasgupta et al., 2009), hepatocellular (Li et al., 2019), gastric (Tu et al., 2016), lung (Davis et al., 2009; Wang and Hu, 2018), and glioblastoma (Pucci et al., 2021) tumor cells. Activation of α7-nAChR also reduces efficiency of chemotherapy in lung, oral, breast, and gastric cancers (Tu et al., 2016; Cheng et al., 2020; Afrashteh Nour et al., 2021). This receptor can form complexes with receptor tyrosine kinases (RTKs) such as epidermal growth factor receptor (EGFR) and platelet-derived growth factor receptor (PDGFR) and inositol-1,4,5-trisphosphate 3-kinase (IP3K) (Chernyavsky et al., 2015; Bychkov et al., 2021). Inhibition of α7-nAChR reduces tumor cell proliferation, migration, and angiogenesis *in vitro* and *in vivo* (Grozio et al., 2008; Brown et al., 2012; Yan et al., 2017; Bu et al., 2019). One of the best studied inhibitors of α7-nAChR are three-finger snake α-neurotoxins (Vasilyeva et al., 2017; Kulbatskii et al., 2018). Their efficacy on tumor growth inhibition *in vivo* was demonstrated on example of α-cobratoxin (Grozio et al., 2008), but such toxins inhibit α7-nAChR irreversibly and with high affinity, that may lead to high systemic toxicity in clinic.

In contrast, some endogenous human proteins that share three-finger fold with snake neurotoxins reversibly modulate α7-nAChR and may serve as prototypes for specific and non-toxic α7-nAChR-targeting drugs. For example, human SLURP-1, a selective negative allosteric modulator of α7-nAChR (Lyukmanova et al., 2016), inhibits growth of different carcinoma and glioma cells *in vitro* in 2D and 3D tumor models (Lyukmanova et al., 2014; 2018; Throm et al., 2018; Bychkov et al., 2019; 2021; Shulepko et al., 2020a; 2023) and abolishes the nicotine-induced cell proliferation (Shulepko et al., 2020b). SLURP-1 controls growth and migration of lung adenocarcinoma A549 cells via interaction with α7-nAChR heterocomplexes with EGFR or PDGFR and modulation of the PI3K/AKT/mTOR and inositol-1,4,5-trisphosphate (IP3) pathways (Shulepko et al., 2020b; Bychkov et al., 2021). One hour incubation of human epidermoid carcinoma A431 cells with recombinant SLURP-1 induces secretion of endogenous SLURP-1 from an intracellular depot, thus increasing the SLURP-1 concentration in extracellular media and overall antiproliferative effect (Lyukmanova et al., 2018). SLURP-1 expression is downregulated in primary and metastatic melanomas compared to normal cells (Bergqvist et al., 2018; Arousse et al., 2019), while elevated plasma level of SLURP-1 correlates with better survival prognosis for patients with pancreatic cancer (Throm et al., 2018). Thus, increased level of SLURP-1 in the blood or other tissues of the body can be considered a promising strategy for cancer therapy.

Recently, loop I of SLURP-1 has been identified as the active site responsible for the antitumor activity of the protein (Bychkov et al., 2021; Shulepko et al., 2021). Here, we investigated the molecular mechanisms underlying SLURP-1 activity in A431 cells *in vitro* and studied the activity of the protein and 21 a.a. peptide mimicking its loop I (named “Oncotag”) *in vivo* in a xenograft mice model of epidermoid carcinoma. Despite that both SLURP-1 and Oncotag inhibited tumor growth *in vivo*, only Oncotag demonstrated prolonged downregulation of pro-oncogenic signaling. Affinity extraction showed that in the xenograft tumor, Oncotag interacts only with α7-nAChR, while SLURP-1 also binds EGFR. Thus, prolonged effect of Oncotag was associated with selective targeting of α7-nAChR.

## 2 Materials and methods

### 2.1 Materials, animals and randomization

Recombinant SLURP-1 was produced in *E. coli* as described previously (Shulepko et al., 2013; Lyukmanova et al., 2014). The 21 a.a. Oncotag peptide mimicking the loop I of SLURP-1 (VKAYTCKEPXTSASCRTITRAa, where “X” is norleucine, “a” is *C*-terminal amide form, and cysteine residues form a disulfide bridge, Supplementary Figure S1A) was obtained by chemical synthesis as described previously (Bychkov et al., 2021). The purity and homogeneity of the protein and peptide preparations were confirmed by HPLC, MALDI-MS, and SDS-PAGE. The disulfide bond formation was confirmed in the reaction with Ellman’s reagent (Sigma-Aldrich, St. Louis, United States). The correct folding of the recombinant SLURP-1 and homogeneity of the Oncotag preparation were also confirmed by ^1^H NMR spectroscopy.

Fluorescently-labeled α-bungarotoxin (α-Bgtx)/Alexa-555 was the product of Life Technologies (B35451). α-Bgtx, AG-825, PD98059, SP600125, SB203580, Bay-11-7082, and Go 6983 were the products of Tocris (Bristol, United Kingdom). JSH-23 was the product of SantaCruz (Dallas, United States). S31-201, 285986-31-4 and Xestospongin B were from Calbiochem (San Diego, United States). Doxorubicin was from TEVA (Tel Aviv-Yafo, Israel).

The animals were bred and housed under the standard conditions of the Animal Breeding Facility, BIBCh, RAS (the Unique Research Unit Bio-Model of the IBCh, RAS; the Bioresource Collection—Collection of SPF-Laboratory Rodents for Fundamental, Biomedical and Pharmacological Studies) accredited at the international level by AAALACi. All procedures were performed in accordance with Rus-LASA ethical recommendations approved by the IBCh RAS Institutional Animal Care and Use Committee (protocol #318/2021).

The study was not pre-registered. Mice were allocated to groups using the randomization software available online at (https://www.graphpad.com/quickcalcs/randomize1.cfm).

### 2.2 Cell cultivation and viability assay

Human epidermoid carcinoma A431 cells (ATCC, Manassas, United States) were grown (37°C, 5% CO_2_) in DME medium with phenol red (PanEco, Moscow, Russia), 10% fetal calf serum (Thermo Fisher Scientific, Waltham, United States) and 2 mM L-glutamine (PanEco), abbreviated below as the complete medium. Cells were subcultured at least twice a week.

To study an influence of SLURP-1 and inhibitors of the intracellular signaling pathways on the A431 cell growth, the cells were seeded in 96-well cell culture plates in the complete medium (7.5 × 10^4^ cells/well) and grown for 24 h. Thereafter, the cells were preincubated with 1 µM PD98059, 100 nM SP600125, 1 μM SB203580, 3 nM wortmannin, 1 µM Go 6983, 10 µM Xestospongin B, 10 µM Bay 11-7082, 1 μM JSH-23, 100 µM S31I-201, or 10 µM 285986-31-4 (dissolved in the complete medium from the 100% DMSO stocks) for 1 h, and the cell medium was changed to the medium containing the inhibitors with or without 1 µM SLURP-1 for further incubation during 24 h. The final maximal DMSO concentration in media did not exceed 0.5%. Added DMSO did not influence the cell growth as was established in the additional experiments.

To analyze cell viability, we used the water soluble tetrazolium salt 1 (WST-1) colorimetric test as described earlier (Lyukmanova et al., 2016). Briefly, WST-1 (Santa Cruz) and 1-m-PMS (1-methoxy-5-methylphenazinium methyl sulfate, Santa Cruz) were added to the cells in concentrations of 0.25 mM and 5 μM, respectively, for 1 h, and formation of colored product was measured at 450 nm with background subtraction at 655 nm on Bio-Rad 680 microplate reader (Bio-Rad, Hercules, United States). The data were normalized to an averaged read-out from the control wells containing cells without added compounds.

### 2.3 α7-nAChR knock-down

To block expression of the native α7 receptor, A431 cells were transfected with siRNA to α7-nAChR (α7-siRNA). siRNA duplex was formed by GGA​AGC​UUU​ACA​AGG​AGC​UGG​UCA​A and UUG​ACC​AGC​UCC​UUG​UAA​AGC​UUC​C synthetic oligonucleotides (Synthol, Moscow, Russia) (Al-Wadei et al., 2012). Cells were seeded in 6-well culture plates (1 × 10^5^ cells per well) and grown for 24 h. Then α7-siRNA (1 μg per well) was diluted in 100 μL of transfection buffer (Pan-Biotech, Aidenbach, Germany), incubated for 5 min and mixed with 15 μL of pre-diluted PanFect A-plus transfection reagent (Pan-Biotech). The final mixture was incubated for 30 min and added to A431 cells. The cells were incubated in CO_2_-incubator during 4 h and the cell media was replaced by the fresh complete medium. After 48 h incubation, the cells were detached by Versene solution and subdivided onto the two parts. The first part was incubated with α-Bgtx/Alexa-555, and expression of functional α7-nAChR on the cell membrane was analyzed by flow cytometry. The second part of the cells was seeded in 96-well culture plates (7.5 × 10^4^ cells per well) and incubated with 1 µM SLURP-1 or 1 µM α-Bgtx for 24 h as described above. Cell viability was analyzed by the WST-1 assay.

### 2.4 Phosphorylation analysis

To determine the influence of SLURP-1 and Oncotag on phosphorylation of intracellular kinases, transcription factors, and other signaling molecules in A431 cells or A431/NanoLuc tumors, we used the chemiluminescent Proteome Profiler human phospho-kinase antibody array kit (ARY003C, R&D Systems, Minneapolis, United States). A431 cells were seeded in flasks in the complete medium (5 × 10^5^ cells/flask) and grown for 24 h, treated with 1 µM SLURP-1 for 1 h, and detached using Versene solution. The A431 cells or A431/NanoLuc tumors (4 tumor samples from each group were randomly chosen) were then lysed in the kit’s lysis buffer, and phosphorylation of the proteins was determined according to the manufacturer’s protocol. The chemiluminescence on the array kit membranes was detected with LAS500 imaging system (GE Healthcare, Chicago, United States). The chemiluminescence of spots representing phosphorylated proteins was measured using ImageJ software. The data were normalized to the averaged density of the reference spots.

### 2.5 Tumor xenograft model, treatment strategy, and living mice imaging

To obtain the luminescent A431/NanoLuc cells, the parental A431 cells were transfected with the NanoLuc plasmid as described in (Shipunova et al., 2020) using FuGENE HD transfection reagent (Promega, Madison, United States). Cells stably expressing NanoLuc were selected at 14^th^ day of cultivation in the complete medium with 0.5 μg/mL puromycin (Sigma-Aldrich).

Male BALB/c Nu/Nu mice (22–25 g) were engrafted subcutaneously with 10^7^ A431/NanoLuc cells in 100 μL of 30% Matrigel (Corning, Corning, United States) in a complete culture medium. On the 3^rd^ day after A431/NanoLuc cells engraftment, mice were randomly divided into four groups (initially n = 10), and i.v. injected once a day for ten subsequent days by 100 µL of 0.9% NaCl solution (saline) containing: 1) no additives - control, 2) 10 µg (0.5 mg/kg) of SLURP-1, 3) 2.5 µg (0.125 mg/kg) of Oncotag, 4) 25 µg (1.25 mg/kg) of Oncotag (Supplementary Figure S1B). Some animals died during the experiment (Supplementary Table S1 and Supplementary Figure S4) and were excluded from the analysis.

The primary tumor volume was measured with a caliper and calculated using the formula:

$$V=0.52\times A\times B^{2},\left(A\text{ is the largest diameter and }B\text{ is the smallest diameter}\right).$$

On 3^rd^, 13^th^, and 23^rd^ days after tumor engraftment, tumors were visualized with the IVIS Spectrum CT imaging system (Perkin Elmer, Waltham, United States). Mice were anesthetized with 2% vaporized isoflurane (using RAS-4 Rodent Anesthesia System, Perkin Elmer), and then received 4 µL of furimazine (Nano-Glo Luciferase Assay System, Promega) in 100 µL of saline by intraperitoneal injection. 20 min later, tumors’ bioluminescence was visualized with an open filter on the IVIS Spectrum CT system. Bioluminescence images were acquired with IS 1803N7357 iKon camera (Andor, Belfast, United Kingdom) using 60-s exposure time (f/stop = 1, binning = 8, field of view 13.4 × 13.4 cm) and normalized to photons per second per cm^2^ per steradian (p/sec/cm^2^/sr). Images were acquired and analyzed using Living Image 4.5.5.19626 software (Xenogen, Alameda, United States).

On the 24^th^ day after tumor engraftment, mice were euthanized by cervical dislocation, the tumors were isolated with scalpel and tweezers. The necrotic zones, if available, were separated and the tumor mass was divided into two parts. The first part of the tumor was placed in a 4% paraformaldehyde solution (Applichem, Barcelona, Spain), and the second part and necrotic zones were separated and immediately frozen at −150°C for further analysis.

All of the procedures were approved by the IBCh RAS Institutional Animal Care and Use Committee (protocol #318/2021).

### 2.6 Tumor immunohistochemistry

Fixed tumor fragments were washed by water, dehydrated in ethyl alcohols of increasing concentration, and embedded in paraffin. Paraffin sections (4–5 μm thick) were stained with hematoxylin and eosin (BioVitrum, St-Petersburg, Russia) as described in (Fischer et al., 2008) and examined by conventional light microscope AxioScope.A1 (Carl Zeiss, Jena, Germany). Microphotographs of histological preparations were obtained using a high-resolution Axiocam 305 color camera (Carl Zeiss) and ZEN 2.6 lite software (Carl Zeiss).

### 2.7 Immunogenicity assay

The immunogenicity of SLURP-1 and Oncotag was studied in C57BL mice. The animals were randomly subdivided into six groups. The first three groups (n = 10 in each group) received: 1) i.v. 10 μL of saline every day for 5 days, 2) i.v. 10 μg of SLURP-1 dissolved in 10 μL of saline every day for 5 days, 3) i.p. mixture of 100 µL of complete Freund’s adjuvant (BD Biosciences, New Jersey, United States) and 10 µg of SLURP-1 on the first day of the study. The last three groups (n = 5 in each group) received: 4) i.v. 10 μL of saline every day for 5 days, 5) i.v. 2.5 μg of Oncotag dissolved in 10 μL of saline every day for 5 days, 6) i.p. mixture of 100 µL of complete Freund’s adjuvant and 2.5 μg of Oncotag on the first day of the study. The groups #3 and #6 were considered positive controls of antibodies production in mice. The level of antibodies to SLURP-1 and Oncotag in the blood serum collected from inferior vena cava was determined using direct ELISA with anti-mouse HRP-conjugated antibodies (1:10000, 115-035-003, Jackson Immunoresearch, West Grove, United States). Formation of a colored product was measured at 450 nm on Bio-Rad 680 microplate reader.

### 2.8 Acute toxicity study

The design of the study was based on the recommendations of the Guidelines for the conduct of preclinical drug trials as well as the recommendations of the OECD Guidelines for the testing of chemicals (OECD, 2002; Buryakina and Merkulova, 2017). Female and male ICR mice were randomly divided into three groups, n = 10 (5 female and 5 male mice in each group), and i.v. injected by 100 µL of saline containing: 1) no additives - control, 2) 50 mg/kg of SLURP-1, 3) 12.5 mg/kg of Oncotag. After the administration of the drug solution or saline, the presence and severity of clinical signs of intoxication, body weight, and feed intake were recorded in animals. Animals were observed for 9 days. Body weight, food intake, and the functional observation battery tests, were recorded on the 1^st^ and 9^th^ day after administration. Acute toxicity study consisted of a sequential assessment of the animal behavior in the cage, when picked up, in an open area, as well as the instrumental assessment of sensory, neuromuscular and physiological parameters. Cage examination included assessment of posture, presence of convulsions, tremors, self-harm, eyelid drooping, piloerection, and assessment of fecal status. The handling examination included an assessment of the ease of removing the animal from the cage, the ease of handling, the presence of lacrimation, chromodacreorea, salivation, appearance of animal hair, breath characteristic, and the presence of discharge from the eyes, oral cavity, nose, anus and genitals, condition of the eyes and mucous membranes, the presence of exophthalmos, assessment of muscle tone. Examination in an open area was carried out on the platform of the automated device (Multiconditioning, TSE, Germany), where the animal was placed for 6 min for an automated assessment of locomotor activity and included an assessment of the number of rears, the ability to move, the number of grooming acts, the nature of gait, the presence and severity of disorders gait, the presence of convulsions, tremors, vigilance, stereotypic behavior, and the number of urinations and defecations. Sensory observations included the assessment of the reaction to the approach of an object, the reaction to touch, the reaction to a sharp sound (Startle response), the reaction to tail pinching, the olfactory test, the assessment of the pupillary and blinking reflexes, and the rollover reflex. Neuromuscular observations included several functional tests: the strength of the flexors-extensors of the hind limbs was assessed when pressing and pulling the hind limbs of the animal fixed in the hand; the distance between the hind limbs was assessed by releasing the animal, taken by the tail, in which the soles of the hind limbs were previously stained with a non-toxic dye, from a height of 20 cm. The strength of the fore and hind limbs was assessed using the Grip Strength Meter (Columbus Instruments, United States). As physiological observations, the respiratory rate of animals was assessed on a computerized PowerLab 8/35 device using a Spirometer unit (ADInstruments, United States) and a respiratory head for mice. On the 10th day, the animals were euthanized and necropsied; the organs were examined for the presence of macrodamages, weighed and fixed in 4% formalin (Applichem, United States).

### 2.9 Real-time PCR for mRNA and miRNA detection and miRNA targets prediction

Total mRNA from the tumors and necrotic zones was extracted by the Aurum Total RNA Mini Kit (Bio-Rad) according to the manufacturer’s instructions. cDNA was synthesized using the Mint revertase (Evrogen, Moscow, Russia) with the oligodT primer or miRNA-specific stem-loop primers (Supplementary Table S2). After that, real-time PCR was performed with the primers described in the Supplementary Tables S2, S3, and ready-to-use qPCR mix with the SYBR Green I fluorescent dye (Evrogen). Negative controls contained all the components of the PCR mixture but with cDNA replaced by mRNA gave no signal. All PCR reactions were performed using a Roche Light cycler 96 amplificator (Roche, Basel, Switzerland). PCR reaction was performed in duplicate for every sample and an average was taken for further analysis. Data was analyzed by the ΔCt method (Livak and Schmittgen, 2001) using Light-Cycler 96 SW1.01 software (Roche). Gene expression level was normalized to expression of the housekeeping genes *ACTB*, *GAPDH*, and *RPL13A* for mRNA or housekeeping non-coding RNA *U6* for miRNA analysis.

### 2.10 Affinity purification and western blotting

For investigation of the SLURP-1 and Oncotag targets in A431/NanoLuc tumors, SLURP-1 (1 mg/mL) and Oncotag (1 mg/mL) were coupled to PureProteome magnetic beads (LSKMAGN01, Millipore, Burlington, United States) according to the manufacturer’s instructions and blocked by 500 mM ethanolamine + 5% non-fat dry milk (Sigma-Aldrich). The beads blocked by 500 mM ethanolamine + 5% non-fat dry milk without any coupled protein was used as a negative control. Four tumor samples from control group were randomly chosen for analysis. The tumors (0.05 mg per sample) were homogenized, solubilized in 2% Triton X-100 (A4975, Panreac), and the lysate was diluted 10 times with TBS buffer (100 mM TRIS, 150 mM NaCl, pH 8.0) for incubation with the beads for 16 h at 4°C in TBS. After that, non-specifically bound proteins were sequentially washed out from the beads with TBS, TBS + 1 M NaCl + 0.5% Triton X-100, and TBS + 0.5% Triton X-100. The specifically bound proteins were eluted by 200 mM glycine (pH 2.6), diluted into non-reducing PAGE loading buffer for detection of EGFR and in reducing PAGE buffer for detection of α7-nAChR. Western blotting was performed as described earlier (Bychkov et al., 2022) using primary antibodies (ABIN5611363, Antibodies Online, Aachen, Germany, 1:1000) and secondary antibodies (111-035-003, Jackson Immunoresearch, West Grove, United States, 1:5000) for detection of α7-nAChR, and primary antibodies (sc-120, Santa Cruz, 1:1000) and secondary antibodies (715-035-150, Jackson Immunoresearch, 1:5000) for EGFR detection. The HRP signal was detected by the ECL substrate (Bio-Rad) using the ImageQuant LAS 500 chemidocumenter (GE Healthcare).

For analysis of the SLURP-1 and Oncotag influence on KLF4 expression, 4 tumor samples from each group were randomly chosen. The tumors (0.05 mg per sample) were homogenized, solubilized in 2% Triton X-100, and diluted into reducing PAGE buffer. Western blotting was performed with primary anti-KLF4 antibodies (NBP2-24749, Novus Bio, Centennial, United States, 1:2000) and secondary anti-rabbit antibodies (111-035-003, Jackson Immunoresearch, 1:5000). KLF4 expression was normalized to the total protein content after ponceau S (Sigma-Aldrich) staining, the data were analyzed using the ImageJ 1.53t software (NIH, Bethesda, United States).

### 2.11 Statistical analysis

Data are presented as mean ± SEM. Sample numbers (n) are indicated in the figure legends. Statistical analysis was performed using GraphPad Prism 9.5.0 software (Graphpad software, San Diego, United States). The data were analyzed for normal distribution by a Shapiro-Wilk omnibus normality test. For non-normally distributed data, the Kruskal–Wallis test was used instead of one-way ANOVA. Analysis was done using two-tailed *t*-test, two-tailed *t*-test followed by Holm-Sidak’s *post hoc* test, Kruskal–Wallis test followed by Dunn’s *post hoc* test, one-way ANOVA followed by Dunnett’s or Tukey’s *post hoc* test, and two-way ANOVA followed by Dunnett’s *post hoc* test as indicated in the figure legends. Differences in the groups were considered statistically significant at *p* < 0.05.

## 3 Results

### 3.1 SLURP-1 inhibits growth of A431 cells via α7-nAChR

**FIGURE 1:**
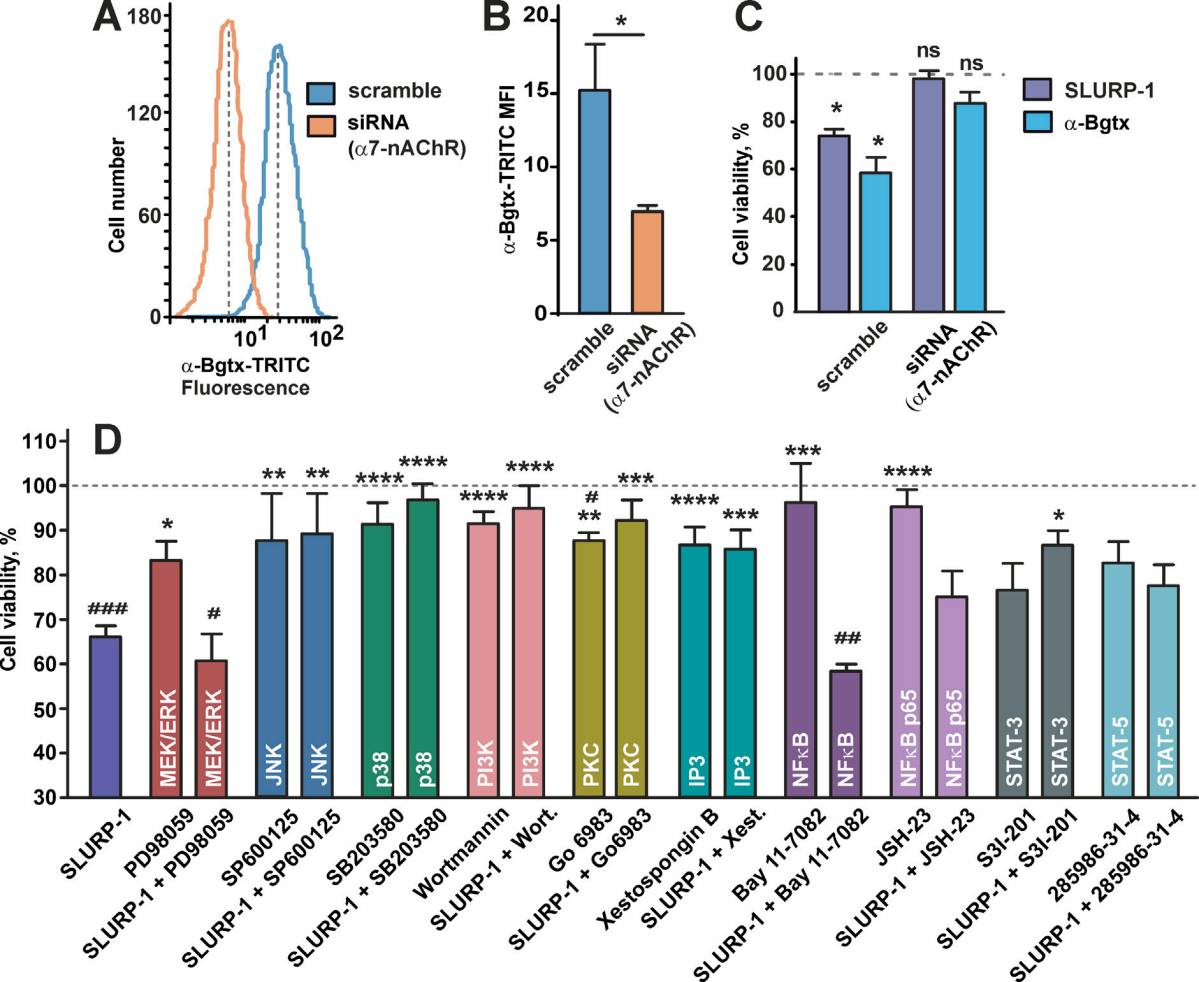
Influence of α7-nAChR knockdown and inhibition of the intracellular signaling pathways or transcription factors on SLURP-1 activity in A431 cells. **(A)** Representative histograms of the cell distribution according to the intensity of TRITC-labeled α-Bgtx for cells transfected with scramble (blue) and with α7-nAChR siRNA (orange). **(B)** Median fluorescence intensities for TRITC-labeled α-Bgtx in cells transfected with scramble (blue) and with α7-nAChR siRNA (orange) (n = 5). * (*p* < 0.05) indicates the significant difference between the groups by two-tailed *t*-test. **(C)** Antiproliferative activity of 1 µM SLURP-1 and 1 µM α-Bgtx in A431 cells transfected with scramble and with α7-nAChR siRNA. Control (100% of viable cells) corresponds to the transfected cells incubated in absence of the proteins. Data presented as % of control ± SEM (n = 6). * (*p* < 0.05) indicates the significant difference from the control by two-tailed one-sample *t*-test. **(D)** Influence of the inhibitors of intracellular signaling pathways and transcription factors on the A431 cell viability in absence and presence of 1 µM SLURP-1. Cells were incubated with SLURP-1 and other compounds as described in the Materials and Methods section. Data presented as % of control ± SEM (n = 3–20). # (*p* < 0.05), ## (*p* < 0.01), and ### (*p* < 0.001) indicate the significant differences from the control (100%) by two-tailed one-sample *t*-test followed by Holm-Sidak’s *post hoc* test; * (*p* < 0.05), ** (*p* < 0.01), and *** (*p* < 0.001) indicate the significant differences from the “SLURP-1” group by One-way ANOVA followed by a Dunnett’s *post hoc* test.

Recombinant SLURP-1 inhibits growth of A431 cells and co-localizes with α7-nAChR presented on the cell membrane (Lyukmanova et al., 2018). To confirm that α7-nAChR is the molecular target of SLURP-1, we downregulated receptor expression by transfecting A431 cells with α7-nAChR siRNA (Figures 1A,B). Incubation of scramble transfected A431 cells with SLURP-1 or snake neurotoxin α-Bgtx (a selective inhibitor of α7-nAChR) significantly decreased cell viability. In contrast, α7-nAChR knockdown abolished antiproliferative effect of both proteins (Figure 1C). Thus, inhibition of A431 cell growth by SLURP-1 is related to α7-nAChR.

### 3.2 SLURP-1 downregulates phosphorylation of different mitogenic kinases and transcription factors in A431 cells

To study the intracellular mechanisms involved in the antiproliferative action of SLURP-1, we investigated the viability of A431 cells upon 24 h incubation with SLURP-1 and selective inhibitors of various signaling pathways: MEK/ERK (PD98059), JNK (SP600125), MAP kinase p38 (SB203580), PI3K (Wortmannin), PKC (Go 6983), IP3 receptors (xestospongin B), and the transcription factors NFκB (Bay 11-7082 and JSH-23), STAT3 (S3I-201), and STAT5 (285986-31-4). The antiproliferative activity of SLURP-1 was blocked by the inhibition of JNK, p38, PI3K, PKC and IP3 (Figure 1D). At the same time, the inhibition of the MEK/ERK kinases and NFkB transcription factor did not influence the SLURP-1 antiproliferative activity, while results for STAT3 and STAT5 transcription factors were uncertain (Figure 1D).

**FIGURE 2:**
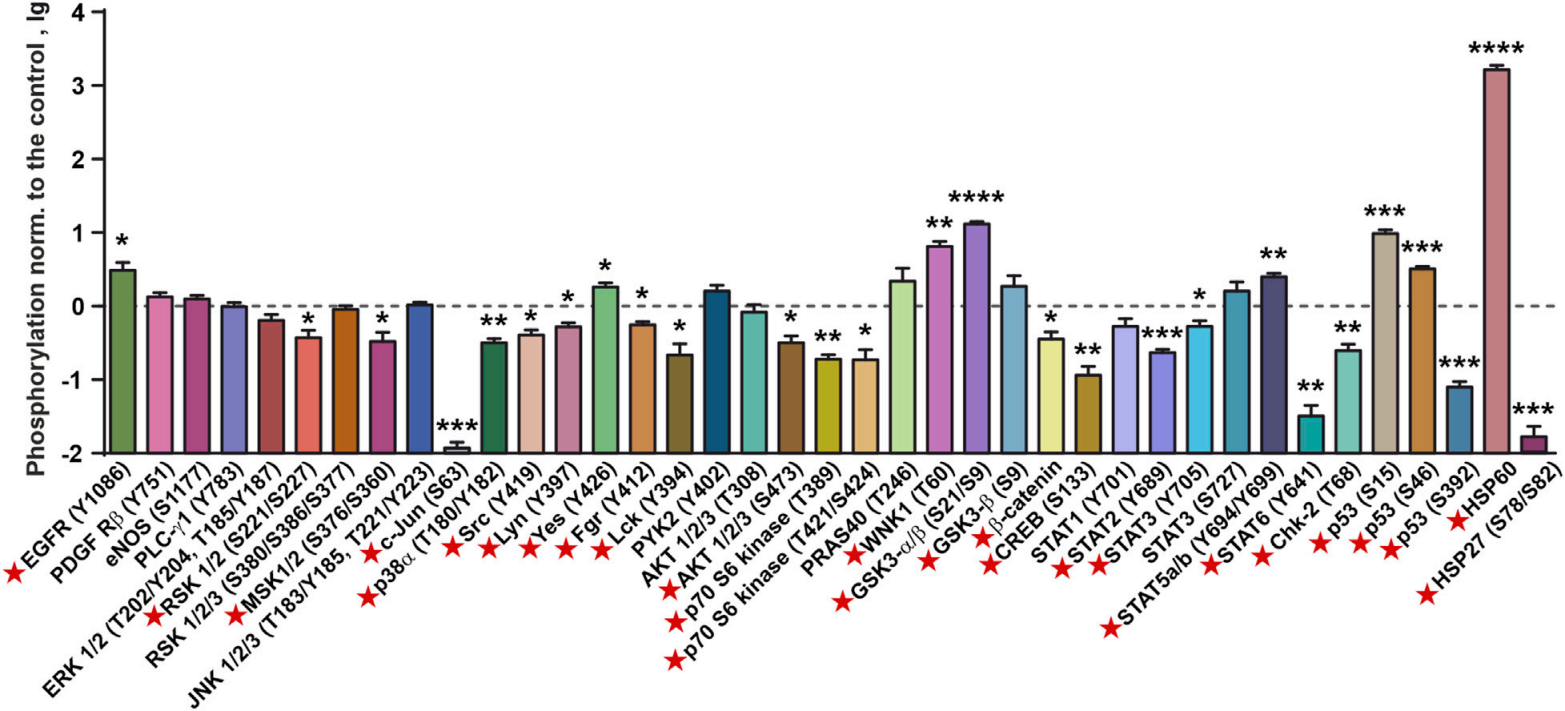
Changes in phosphorylation of the cell surface receptors, intracellular kinases, and regulatory proteins in A431 cells after 1 h incubation with 1 µM SLURP-1. Data are presented as lg of the phosphorylation level normalized to the control (untreated cells, 0, dashed line) ± SEM (n = 4). * (*p* < 0.05), ** (*p* < 0.01), *** (*p* < 0.001), **** (*p* < 0.0001) indicate the significant difference from the control (0, level) according to the two-tailed one-sample *t*-test. Signal molecules for which significant difference of phosphorylation from the control level was revealed are marked by red asterisks. The legend for the spot identification is shown in Supplementary Figure S2 and the original membranes of dot-blot based analysis are shown in Supplementary Figure S3.

Previously, we showed that incubation of A431 cells with recombinant SLURP-1 during 1 h causes secretion of endogenous intracellular SLURP-1 to the extracellular media (Lyukmanova et al., 2018). To describe the mechanisms involved in the early SLURP-1 action, we analyzed the relative changes in phosphorylation of the intracellular kinases and transcription factors (Figure 2; Supplementary Figures S2, S3). One hour incubation with SLURP-1 significantly decreased the phosphorylation of signaling molecules responsible for pro-oncogenic and mitogenic signals: mitogenic kinases RSK 1/2 (S221/S227), MSK 1/2 (S376/S360), c-Jun (S63), AKT 1/2/3 (S473), p70 S6 kinase (T389 and T421/T424), and p38α MAP kinase (T180/Y182), pro-oncogenic Src-family kinases (Src (Y419), Lyn (Y397), Fgr (Y412) and Lck (Y394), which activate tumor cell invasion (Ortiz et al., 2021)), the pro-oncogenic heat-shock protein HSP27 (S78/S82), which inhibits apoptosis and promotes tumor cell survival (Choi et al., 2019), and the WNT-signaling messenger β-catenin (Figure 2). In addition, we observed an increase in the phosphorylation of GSK3-α/β (S21/S9), which is inactivated by AKT and inhibits β-catenin (Duda et al., 2020) (Figure 2). SLURP-1 increased phosphorylation of the transcription factors STAT5a/b (Y694/Y699), probably, activating them, but decreased phosphorylation of CREB (S133), STAT2 (Y689), STAT3 (Y705), and STAT6 (Y641), which are important for tumor progression and regulation of tumor inflammatory microenvironment (Loh et al., 2019) (Figure 2). Increase in phosphorylation of the anti-oncogenic p53 protein at the S15 and S46 positions with the simultaneous decrease of phosphorylation at S392 was also observed (Figure 2). Although, p53 is inactive in A431 cells (Reiss et al., 1992), so its phosphorylation is unlikely to play any physiological role.

From the other hand, SLURP-1 significantly increased phosphorylation of some pro-oncogenic molecules, such as EGFR (Y1086), the Src-family kinase Yes (Y426), and WNK1 kinase (T60), which activates the PI3K/AKT signaling (Gallolu Kankanamalage et al., 2018), and the oxidative stress inhibitor HSP60, which enhances proliferation and inhibits apoptosis (Tang et al., 2022) (Figure 2). SLURP-1 also decreased phosphorylation of the anti-oncogenic kinase Chk-2 (T68), which regulates the cell response to DNA damage and inhibits cell division (Antoni et al., 2007).

### 3.3 SLURP-1 and its peptide mimetic Oncotag inhibit tumor growth in xenograft model

Previously, we have shown that the synthetic 21 a.a. Oncotag peptide mimicking the loop I of SLURP-1 stabilized by intramolecular disulfide bond inhibited growth and migration of A549 cells via selective interaction with α7-nAChR (Bychkov et al., 2021). To study the antitumor effect of SLURP-1 and Oncotag *in vivo*, we used xenograft mice model of human epidermoid carcinoma. A431 cells stably expressing NanoLuc luciferase activated by furimazine were used. This approach allows a bioluminescence imaging of tumor progression and possible metastasis *in vivo*.

The robust antiproliferative effect of SLURP-1 in A431 cells was observed at the concentration of 1 µM (Lyukmanova et al., 2018) (Figures 1C,D). To achieve the similar SLURP-1 concentration in the blood of an animal (mouse, weight −20 g, blood volume −1 mL), the protein was administered intravenously at a dose of 10 μg per mouse (−0.5 mg/kg) (Supplementary Figure S1B). Due to the lower molecular weight of the peptide, we used the Oncotag dose of 0.125 mg/kg, which is similar in molarity to the 0.5 mg/kg dose of SLURP-1. Also, we tested 10 times larger Oncotag dose (1.25 mg/kg).

**FIGURE 3:**
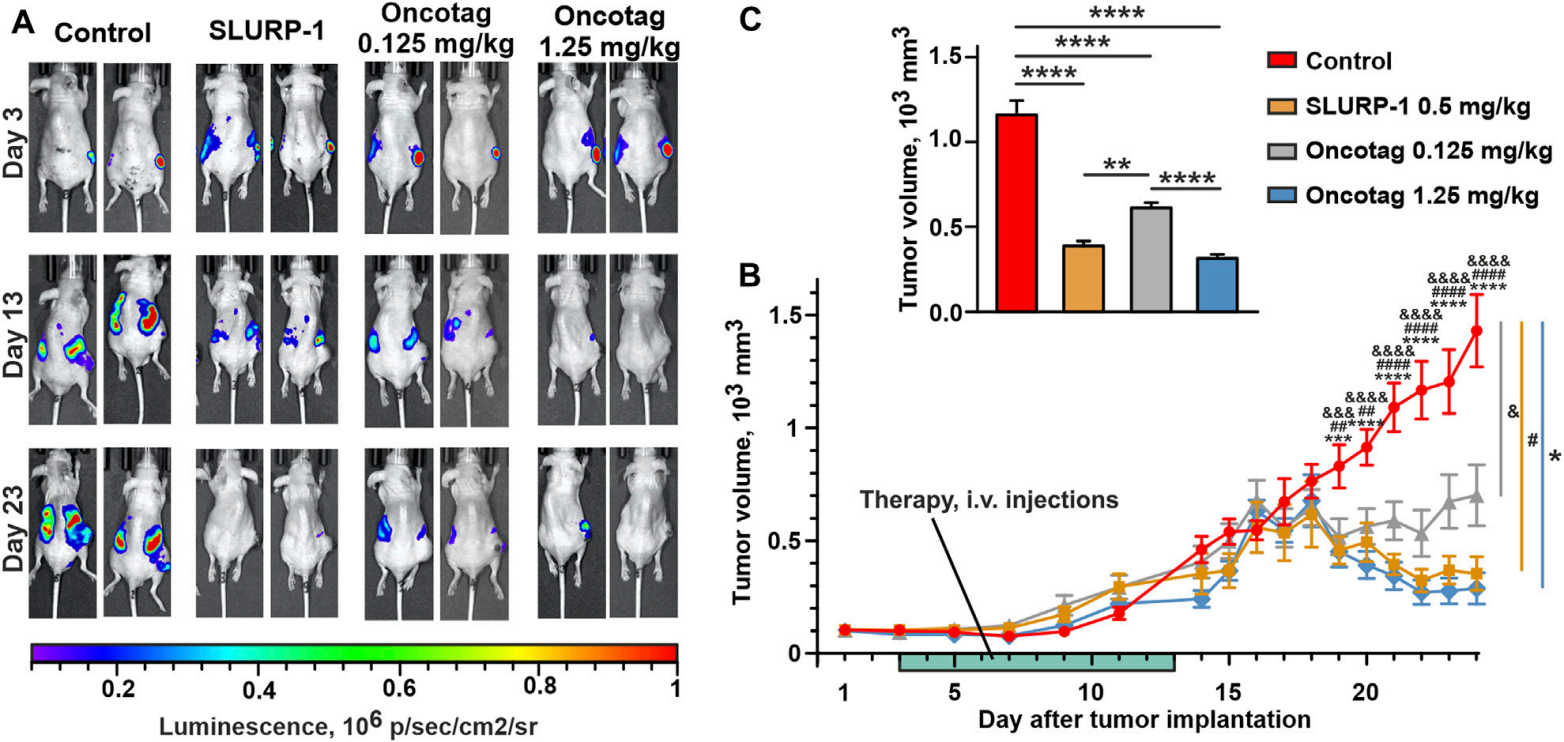
Influence of SLURP-1 and Oncotag administration on the tumor growth in A431/NanoLuc mice xenograft model. **(A)** Representative images of tumor bioluminescence (A431/NanoLuc cells) before treatment (3^rd^ day after tumor engraftment, 1^st^ day of the therapy), after treatment (13^th^ day after tumor engraftment, the next day after end of the 10-day therapy), and before sacrification (23^rd^ day after tumor engraftment). **(B)** The primary tumor volume measurements with a caliper. Data presented as mm^3^ ± SEM. *** (*p* < 0.001) and **** (*p* < 0.0001) indicate the significant difference between “Control” and ’Oncotag 1.25 mg/kg’ groups; #### (*p* < 0.0001) indicates the significant difference between ‘Control’ and “SLURP-1” groups; && (*p* < 0.01), &&& (*p* < 0.001) and &&&& (*p* < 0.0001) indicate the significant difference between “Control” and ‘Oncotag 0.125 mg/kg’ groups according to two-way ANOVA followed by the Dunnett’s *post hoc* test. The days of treatment are marked with light green. **(C)** The average of primary tumor volume measured with a caliper last 5 days (20–24 days after tumor engraftment). Data presented as mm^3^ ± SEM. * (*p* < 0.05) and **** (*p* < 0.0001) indicate the significant difference between groups according to one-way ANOVA followed by a Tukey’s *post hoc* test.

The administration with SLURP-1 or Oncotag at both doses of tumor-bearing mice once a day for 10 subsequent days significantly inhibited primary tumor growth beginning from the 17^th^ day after start of the therapy (the 19^th^ day after tumor engraftment, Figures 3A–C, Supplementary Figure S4, S5A) and resulted in 2-4-fold reduction in the primary tumor volume compared to the control mice (treated with saline) at the 20–24^th^ days after tumor engraftment (Figure 3C). The effect of Oncotag at 1.25 mg/kg was stronger than at 0.125 mg/kg and was comparable to that of SLURP-1 at 0.5 mg/kg. Using bioluminescence imaging, massive distant metastasis in the control mice was revealed (Figure 3A and Supplementary Figure S4). The administration of SLURP-1 and Oncotag at the high concentration significantly suppressed metastasis growth (Figure 3A and Supplementary Figure S5). The administration with low concentration of Oncotag also demonstrated tendency to inhibition of metastasis growth, although changes didn’t reach statistical significance (Figure 3A and Supplementary Figure S5).

Notably, the therapy with SLURP-1 and Oncotag resulted in the formation of necrotic core in tumors, not observed in the control mice (Supplementary Figure S6A). The histological examination revealed that the necrotic core was surrounded by living tumor cells and a mononuclear interlayer, and no morphological differences were found in tumors between the experimental and control groups (Supplementary Figures S6B–D).

### 3.4 SLURP-1 and Oncotag demonstrate no acute toxicity and low immunogenicity

To study acute toxicity, SLURP-1 was administered to mice at a dose of 50 mg/kg (1 mg of the protein in 100 µL of saline), that was 100 times higher than the therapeutic dose of the protein. Due to solubility limitations, the dose of Oncotag was 12.5 mg/kg (0.25 mg in 100 µL of saline), that was 10 times higher than the therapeutic dose of the peptide. SLURP-1 and Oncotag did not cause mortality, had no toxic effects, and did not affect organ weight. Although, SLURP-1 reduced the pupillary reflex on the 2^nd^ day after administration and increased locomotor activity on the 9^th^ day after administration only in males, which indicates a greater sensitivity to SLURP-1 in male ICR mice compared to females. In female mice, only decreased body weight gain was observed in the first day after SLURP-1 administration. Oncotag reduced locomotor activity on the 2^nd^ day of the study and increased body weight gain on the 9^th^ day only in male mice. The observed effects were of a minor nature and did not have a critical impact on the condition of the animals.

We didn’t study the chronic toxicity of SLURP-1 and Oncotag, but note that some animals died during the experiment on tumor treatment (Supplementary Table S1 and Supplementary Figure S4). In the most cases, the cause of death is not clear. However, the number of dead mice in the control group was greater (−30%) than in the SLURP-1 and Oncotag groups (10%–20%), although this difference is not statistically significant. In addition, we also noted that all deaths in the control, SLURP-1, and Oncotag groups occurred on days 16–24, i.e., already after the end of treatment. So, most likely, these deaths are associated with tumor growth and metastases, and not directly with the treatment. On the other hand, the presence of some chronic toxicity of SLURP-1 and Oncotag cannot be excluded and should be studied in future (see Discussion).

Intravenous administration for 5 subsequent days of SLURP-1 or Oncotag at therapeutic doses of 0.5 mg/kg and 0.125 mg/kg (low therapeutic dose), respectively, did not cause an immune response in C57/6J mice (Supplementary Figure S7). Although the immunogenicity of Oncotag at high therapeutic dose and chronic toxicity have not been tested, it can be concluded that the administration of SLURP-1 and Oncotag is safe for at least 10 days of treatment.

### 3.5 SLURP-1 and Oncotag administration increases *CHRNA7*, *CERK*, *KLF4*, and *SLURP1* expression in A431 tumors in mice

**FIGURE 4:**
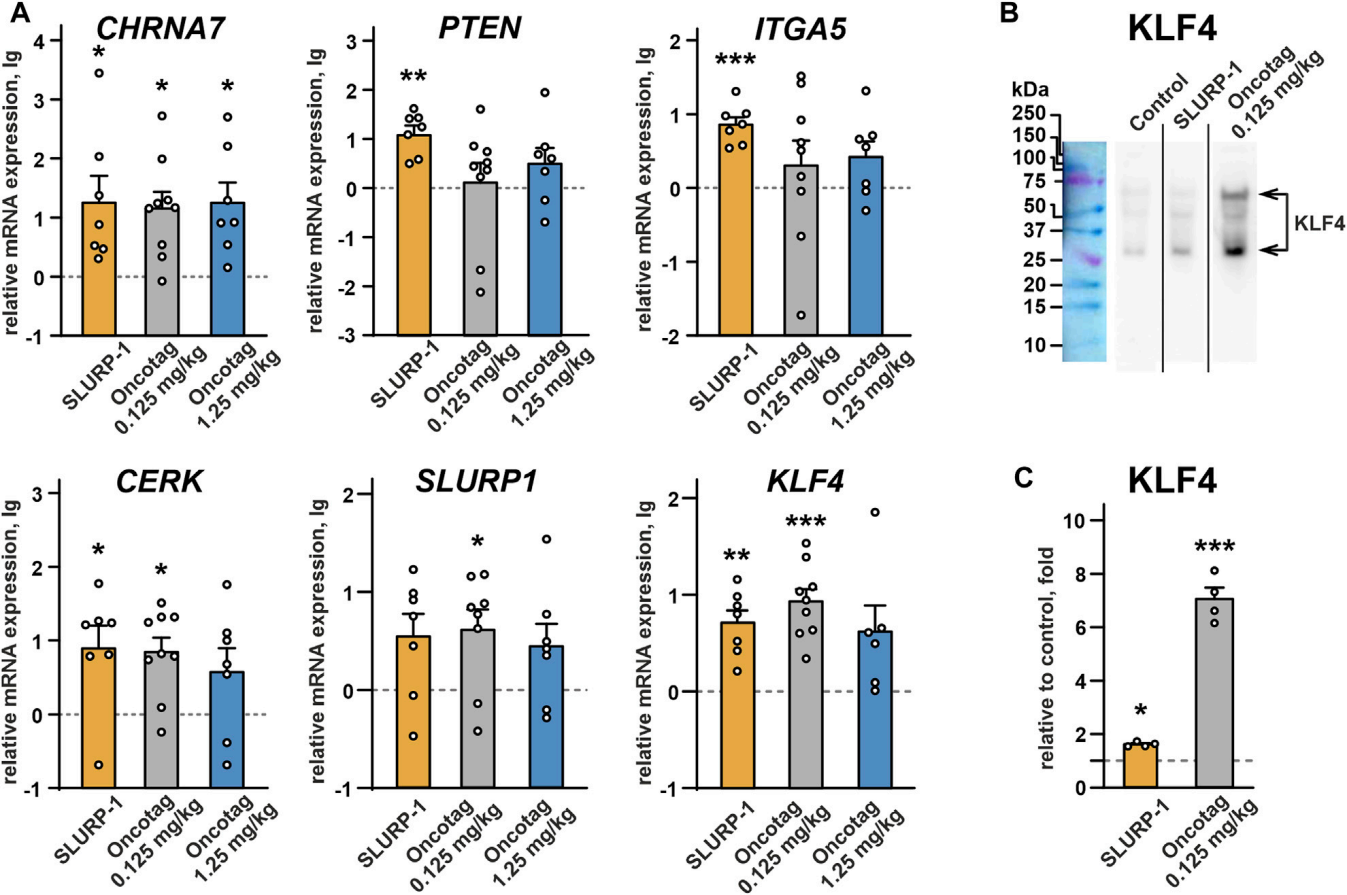
**(A)** Real-time PCR analysis of the mRNA expression of genes coding α7-nAChR (*CHRNA7*), PTEN (*PTEN*), integrin α5 (*ITGA5*), ceramide kinase (*CERK*), SLURP-1 (*SLURP1*), KLF4 (*KLF4*) in mice xenografted tumors after the 10-day treatment with SLURP-1 (0.5 mg/kg), Oncotag (0.125 mg/kg), or Oncotag (1.25 mg/kg) and 11 subsequent days of rest. Data are presented as lg of the mRNA expression level normalized to the expression of the same gene in the control group (mice treated with saline, 0, dashed line) ± SEM (n = 7–9). For each sample the gene expression was pre-normalized to expression of *ACTB*, *GAPDH*, and *RPL13A* genes of housekeeping proteins. * (*p* < 0.05), ** (*p* < 0.01), and *** (*p* < 0.001) indicate significant differences from control group (0, level), by a two-tailed one-sample *t*-test, followed by the Holm-Sidak’s *post hoc* test. **(B)** Representative Western blot membrane with analysis of the KLF4 expression in tumors after the saline (control), SLURP-1, and Oncotag (0.125 mg/kg) treatment. Whole membranes are in Supplementary Figure S7. **(C)** KLF4 expression on a protein level normalized to control (saline, 1, dashed line) group ± SEM (n = 4). The KLF4 level on each blot was pre-normalized to the total protein level according to ponceau S staining (Supplementary Figure S7). * (*p* < 0.05) and *** (*p* < 0.001) indicate significant difference from the control (1, level) by one-sample two-tailed *t*-test followed by Holm-Sidak’s *post hoc* test.

To study processes and intracellular signaling cascades activated upon the SLURP-1 or Oncotag administration in mice, we analyzed gene expression in the A431/NanoLuc tumors and necrotic zones at the 24^th^ day after tumor engraftment *post mortem*. In tumors of SLURP-1 and Oncotag treated mice we did not find any changes in expression of the genes coding EGFR (*EGFR*), PDGFR-α (*PDGFRA*), beta-catenin 1 (*CTNNB1*), integrins α2, α3, and αV (*ITGA2, ITGA3* and *ITGAV*, respectively), vascular endothelial growth factor A (*VEGFA*), activating transcription factor ATF2 (*ATF2*), oncogene c-MYC (*MYC*), macrophage migration inhibitory factor MIF (*MIF*), monooxygenase activation protein YWHAZ (*YWHAZ*), and cyclin dependent kinase inhibitor p27 (*CDKN1B*) relative to the control mice (Supplementary Figure S8). However, in tumors of the SLURP-1-treated mice, we observed a significant increase in expression of the genes coding α7-nAChR (*CHRNA7*), pro-oncogenic integrin α5 (*ITGA5*), and ceramide kinase (*CERK*), which are responsible for growth and migration of cancer cells (Morozevich et al., 2012; Grando, 2014; Camacho et al., 2022) (Figure 4A). At the same time, the SLURP-1 treatment resulted in increased expression of *PTEN,* coding the anti-oncogenic negative regulator of the AKT-PI3K signaling pathway PTEN (Chalhoub and Baker, 2009), and *KLF4*, coding the kruppel-like factor 4 (KLF4) critical for differentiation of epithelial cells (Vangapandu and Ai, 2009). Oncotag treatment increased expression of *CHRNA7*, *CERK*, and *KLF4,* but did not influence *ITGA5* and *PTEN* expression (Figure 4A). Notably, KLF4 regulates expression of different genes, including *SLURP1* (Vangapandu and Ai, 2009; Swamynathan et al., 2012). Indeed, in tumors after the SLURP-1 or Oncotag treatment, we observed the similar increase in *SLURP1* gene expression, but this effect reached statistical significance only in the Oncotag 0.125 mg/kg group (Figure 4А). Western blot analysis confirmed KLF4 increased expression on a protein level with more pronounced effect after the Oncotag treatment (Figures 4B,C).

The real-time PCR analysis of the necrotic zones revealed the effects similar to those observed in the tumor samples (Supplementary Figure S8). Although, the necrotic zones had very small thickness and their samples could be contaminated with surrounding tumor tissues and *vice versa* (Supplementary Figure S6).

### 3.6 Oncotag, but not SLURP-1, sustainably reduces phosphorylation of pro-oncogenic messengers in tumors

The tumor volume after the treatment with high dose of Oncotag (1.25 mg/kg) was very small and it was very difficult to separate the tumor from the necrotic zones (Supplementary Figure S6). At the same time, the effects on gene expression observed in tumors treated by different concentrations of Oncotag were comparable (Figure 4 and Supplementary Figure S8). Therefore, for further analysis, we chose the tumor samples obtained after the treatment by the low dose (0.125 mg/kg) of Oncotag.

**FIGURE 5:**
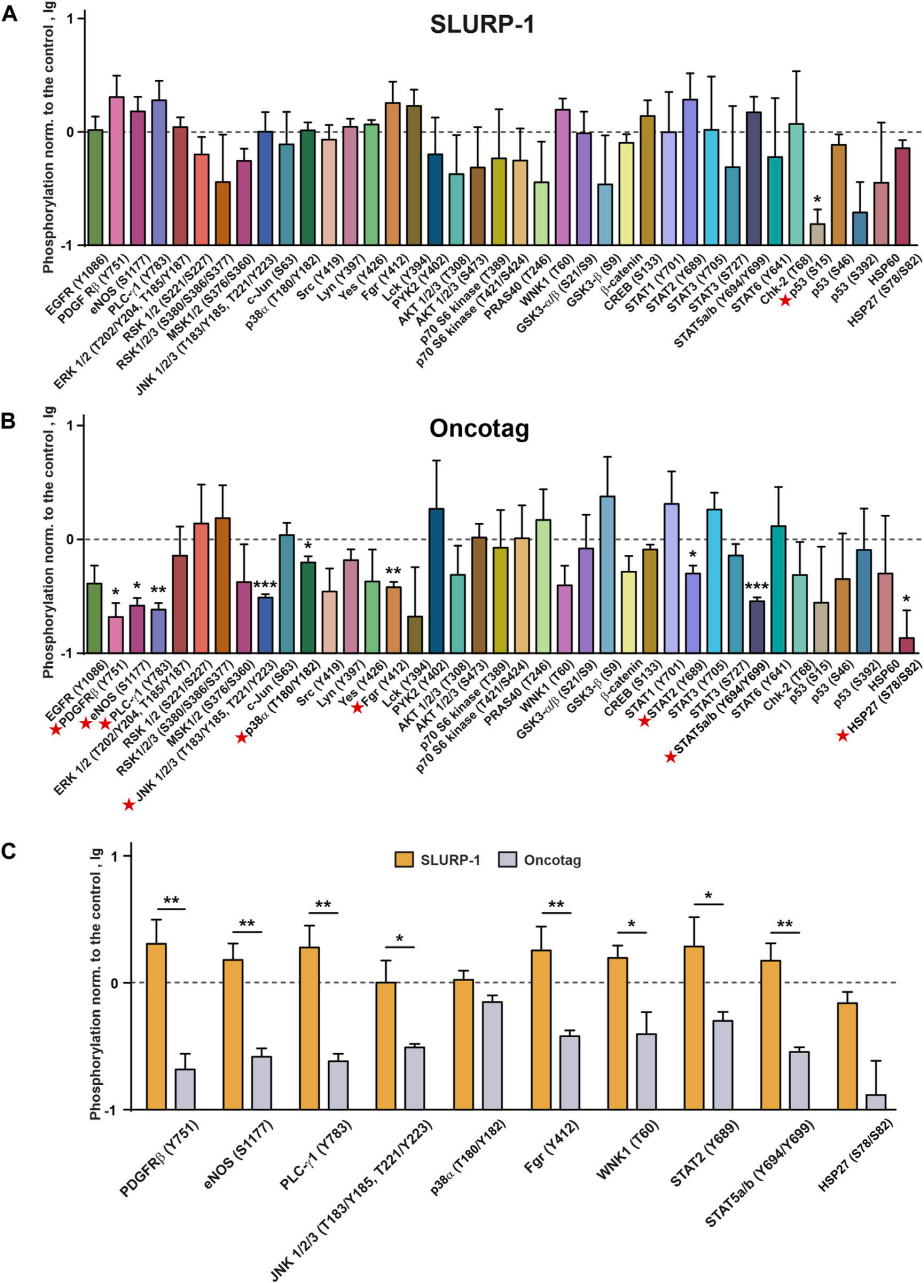
Phosphorylation of the cell surface receptors, intracellular kinases, and regulatory proteins in mice xenografted tumors after the 10-day treatment with SLURP-1 (0.5 mg/kg) **(A)** or Oncotag (0.125 mg/kg) **(B)** and 11 subsequent days of rest. Data are presented as lg of the phosphorylation level normalized to the phosphorylation level of the same protein in the control group (mice treated with saline, 0, dashed line) ± SEM (n = 4). * (*p* < 0.05), ** (*p* < 0.01) and *** (*p* < 0.001) indicate the significant difference from the control (0, level) according to the two-tailed one-sample *t*-test. Signal molecules for which significant difference of phosphorylation from the control level was revealed are marked by red asterisks. **(C)** Comparison of the phosphorylation levels of the selected kinases and regulatory proteins in the tumor samples after the SLURP-1 and Oncotag treatment (data are taken from the panels A and B). * (*p* < 0.05) and ** (*p* < 0.01) indicate significant difference between the data groups according to the two-tailed *t*-test. Please note the difference in the vertical scales between Figures 2, 5.

In contrast to the data obtained in A431 cells upon 1 h incubation with SLURP-1 (Figure 2), no significant changes in phosphorylation of the mitogenic kinases were found in mice xenografted tumors after 10 days of the SLURP-1 administration and 11 subsequent days of rest (Figure 5A). At the same time, the Oncotag treatment resulted in the significant decrease of phosphorylation of PDGFRβ (Y751), the antioxidant enzyme eNOS (S1177), RTK messenger PLC-γ1 (Y783), mitogenic kinases JNK 1/2/3 (T183/Y185, T221/Y223) and p38α (T180/Y182), Src-family kinase Fgr (Y412), transcription factors STAT2 (Y689) and STAT5a and STAT5b (Y694/Y699), and apoptosis inhibitor HSP27 (S78/S82) (Figure 5B). The observed decrease of phosphorylation levels is more evident upon a direct comparison of the tumors of SLURP-1 and Oncotag treated mice (Figure 5C).

### 3.7 SLURP1 treatment decreases miR-7, miR-31, miRNA-135b, miR-203, and miR-221 expression, while Oncotag increases miR-203 expression

**FIGURE 6:**
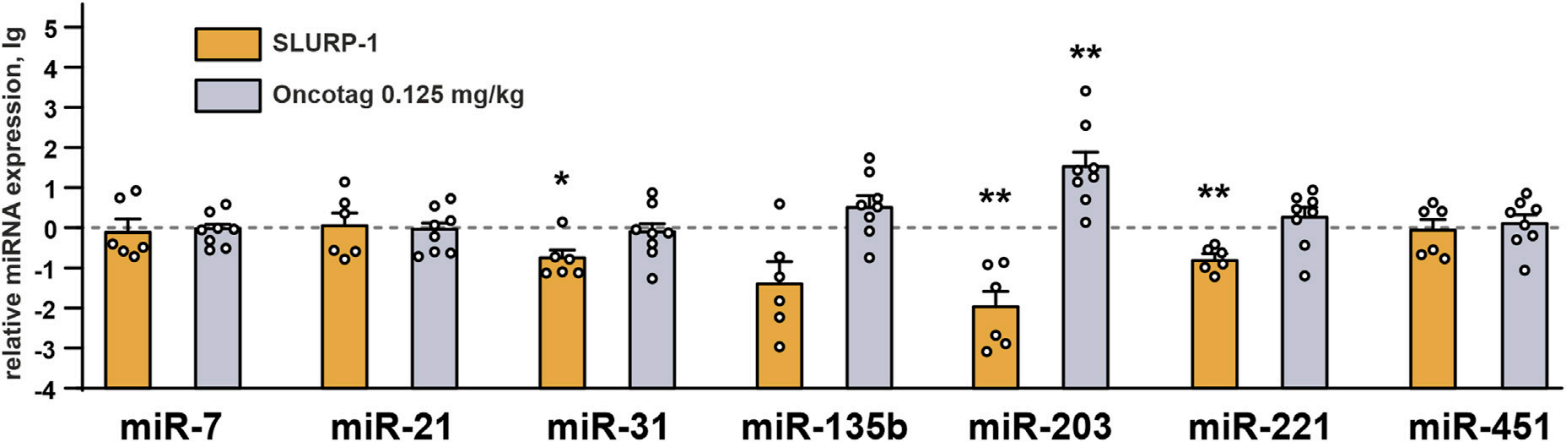
miRNAs expression in mice xenografted tumors after the 10-day treatment with SLURP-1 (0.5 mg/kg) and Oncotag (0.125 mg/kg) and 11 subsequent days of rest. Data are presented as lg of the miRNA expression level normalized to the expression of the same miRNA in the control group (mice treated with saline, 0, dashed line) ± SEM (n = 6–9). For each sample the miRNA expression was pre-normalized to the expression of U6 gene. * (*p* < 0.05), ** (*p* < 0.01), and *** (*p* < 0.001) indicate the significant difference from the control (0, level) by one-sample two-tailed *t*-test followed by Holm-Sidak’s *post hoc* test.

As SLURP-1 and Oncotag influenced expression of some genes in the A431/NanoLuc tumors (Figure 4), we investigated whether it could also affect expression of miRNAs involved in skin cancer progression (Neagu et al., 2020): tumor suppressive miR-7 and miR-203 (Sonkoly et al., 2012; Horsham et al., 2015, 7), pro-oncogenic miR-21, miR-135b, and miR-221 (Yang et al., 2011; Garofalo et al., 2012; Hu et al., 2019), and miR-31 and miR-451 with context-dependent action on tumor growth (Wang et al., 2014; Yu et al., 2018; Bai and Wu, 2019; Fu et al., 2021). The SLURP-1 treatment significantly decreased expression of pro-oncogenic miR-221, as well as of tumor suppressive miR-203, and controversial miR-31 (Figure 6). In contrast, Oncotag increased expression of miRNA-203 only (Figure 6). The upregulation of this miRNA in cutaneous squamous cell carcinoma (CSSC) and melanoma is associated with a better prognosis (Wang and Zhang, 2015; Cañueto et al., 2017). Therefore, SLURP-1 action on miRNAs expression has mixed pro/anti-oncogenic effect, while the Oncotag treatment can make tumors less aggressive.

### 3.8 Oncotag binds only α7-nAChR in A431/NanoLuc tumors, while SLURP-1 also interacts with EGFR

**FIGURE 7:**
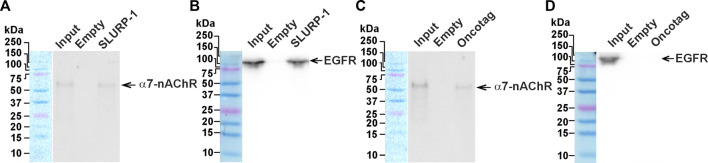
Analysis of the SLURP-1 and Oncotag targets in miсe xenografted tumors. Magnetic beads coupled with SLURP-1 or Oncotag were incubated with a total lysate of tumor samples from mice treated by saline, and extracted proteins were analyzed by Western blotting using antibodies against α7-nAChR **(A and C)** and EGFR **(B and D)**. For detection of EGFR, non-reducing SDS-PAGE was used. Lines: “Input”– the total lysate of tumor sample used for analysis; “Empty”– proteins extracted from the membrane fraction by empty magnetic beads without SLURP-1 or Oncotag; “SLURP-1” and “Oncotag”–proteins extracted from the total lysate by magnetic beads coupled with SLURP-1 or Oncotag. Bands corresponding to the EGFR and α7-nAChR are shown by arrows. Whole Western blot membranes are in Fig. S10.

We have previously shown that SLURP-1 inhibits migration of lung adenocarcinoma A549 cells via interaction with α7-nAChR/EGFR/PDGFR complexes, while the anti-migration activity of Oncotag in A549 cells depends solely on interaction with α7-nAChR (Bychkov et al., 2021). Here, we extracted the molecular targets of SLURP-1 and Oncotag from xenografted A431/NanoLuc tumors of the mice treated by saline using magnetic beads coupled with SLURP-1 or Oncotag. Western blot analysis showed that SLURP-1 extracted both α7-nAChR and EGFR from the tumor homogenate (Figures 7A,B), while Oncotag bound only α7-nAChR (Figures 7C,D).

## 4 Discussion

SLURP-1 was described as an allosteric inhibitor of α7-nAChR (Lyukmanova et al., 2016), but downstream signaling pathways underlying its antiproliferative activity in various cells were not clear (Lyukmanova et al., 2018). Here, we confirmed the association of SLURP-1 antiproliferative effect in epidermoid carcinoma A431 cells with α7-nAChR (Figures 1A–C) and showed its dependence on mitogenic signaling pathways, Src family kinases and STAT transcription factors (Figures 1D, 2). Surprisingly, the SLURP-1 effect was also associated with activation of several pro-oncogenic molecules including EGFR, Src-family kinase Yes and WNK1 kinase, which activate the PI3K/AKT signaling (Gallolu Kankanamalage et al., 2018). Thus, the overall SLURP-1 antiproliferative activity results from an interplay between several pro-oncogenic and anti-oncogenic intracellular signaling pathways.

Intravenous 10-day administration with SLURP-1 or its peptide mimetic Oncotag of mice with A431/NanoLuc xenografted tumors resulted in inhibition of primary tumor and metastases growth (Figure 3 and Supplementary Figures S4, 5). In addition to similar antitumor effects of SLURP-1 and Oncotag *in vivo*, they both led to increase of *CHRNA7*, *CERK*, *KLF4*, and *SLURP1* expression in A431/NanoLuc tumors on 12^th^ day after the end of the therapy. However, increased *ITGA5* and *PTEN* expression in tumors was observed only in the case of SLURP-1 (Figure 4). Increased *CHRNA7* and *ITGA5* expression correlates with poor prognosis in patients with various types of cancer including epidermoid carcinoma (Morozevich et al., 2012; Yang et al., 2015; Hou et al., 2020; Wang et al., 2021), while elevated *CERK* expression correlates with breast tumor recurrence (Payne et al., 2014). Thus, the simultaneous increase in *CHRNA7*, *CERK* and *ITGA5* expression upon the SLURP-1 treatment illustrates the activation of pro-oncogenic intracellular signaling pathways in the tumor. On the other hand, increased *PTEN* and *SLURP1* expression (Figure 4) could neutralize the negative effects of increased *CHRNA7* expression. Indeed, PTEN is a negative regulator of the PI3K/AKT signaling pathway (Chalhoub and Baker, 2009) activated by α7-nAChR (Grando, 2014), and SLURP-1 is the direct inhibitor of α7-nAChR (Lyukmanova et al., 2016).

KLF4 loss leads to increased tumor cell growth in skin cancer (Li et al., 2012). Here, we observed increased KFL4 expression in tumors of mice treated with SLURP-1 or Oncotag both on gene and protein levels (Figures 4A–C). SLURP-1 treatment resulted in 50% increase of KLF4 expression, while Oncotag increased it by −7 times (Figure 4C)*.* KLF4 expression is mediated by the PKC kinase (Chew et al., 2011), which is involved in the SLURP-1 action (Figure 1D) and probably in the Oncotag action via the PLC-γ1/PKC signaling (Figure 5B). Moreover, KLF4 can induce *SLURP1* expression by regulating its promoter (Swamynathan et al., 2012) and cause upregulation of miR-203 expression (Xu Q. et al., 2016). Indeed, we observed increased *SLURP1* and miR-203 expression in tumors upon the Oncotag treatment (Figures 4, 6). Thus, increased *PTEN*, *KLF4*, and *SLURP1* expression points on activation of anti-oncogenic pathways in the tumor.

SLURP-1 and Oncotag differently affected miRNA expression in tumors (Figure 6). SLURP-1 therapy resulted in downregulation of oncogenic miRNA-221 (Figure 6), which targets *PTEN* and is overexpressed in CSSCs (Gong et al., 2019). Thus, upregulation of *PTEN* expression upon the SLURP-1 treatment could be a result of the miR-221 inhibition (Figures 4, 6). Similarly, increased *ITGA5* expression could be a result of downregulated miR-31 (Xu T. et al., 2016) (Figures 4, 6). Simultaneous downregulation of prooncogenic miR-221, tumor suppressive miR-203, and context-dependent tumor suppressive/pro-oncogenic miR-31 (Figure 6) additionally highlights the dual effect of SLURP-1 in cancer cells. In contrast, Oncotag upregulated expression of only miRNA-203 (Figure 6), which leads to less aggressiveness of skin carcinomas (Cañueto et al., 2017). Notably, miR-203 suppresses the PI3K/AKT pathway (Liang et al., 2015; Han et al., 2020), which could be activated by α7-nAChR (Grando, 2014). Thus, miR-203 overexpression could neutralize negative effect of *CHRNA7* overexpression observed under the Oncotag treatment.

Contrarily to the situation observed after 1-h incubation of A431 cells with SLURP-1 (Figure 2), the analysis of phosphorylation of signaling proteins in the xenografted A431/NanoLuc tumors after 10-day therapy with SLURP-1 and 11 days of rest did not reveal significant effects compared to the control (Figure 5A). In contrast, the Oncotag treatment resulted in sustained downregulation of the pro-oncogenic molecules such as PDGFRβ, eNOS, PLC-γ1, JNK, p38α, Fgr, transcription factors STAT2, STAT5, and anti-apoptosis factor HSP27 (Figure 5B) and, probably, in long-term decrease in tumor aggressiveness. Indeed, JNK inactivation upon the Oncotag treatment may block the negative effects of increased *CERK* expression (Gangoiti et al., 2008). Due to possible suppression of the negative effects from α7-nAChR overexpression by miRNA-203 upregulation discussed above, the p38α kinase activated by α7-nAChR (King et al., 2017) can also become blocked in the tumors upon the Oncotag treatment.

One of the most surprising findings of the present study is that tumor volume began to decrease only on the 5^th^ day after the end of the 10-day therapy (Figure 3), and gene expression changes in tumors persisted even after 11 days of rest (Figure 4). Probably, SLURP-1 and Oncotag reprogrammed the tumor cells, which stop their proliferation and stimulated apoptosis or necrosis, and these effects persisted for several days. Indeed, the necrosis regions were formed in the middle of the primary tumors (Supplementary Figure S6), where the environment became deficient in nutrients and oxygen. The exact mechanism of tumor cell death under the action of SLURP-1 or Oncotag requires additional investigation. The long-term action of SLURP-1 and Oncotag can also be explained by the previously observed effect, when incubation of A431 cells with recombinant SLURP-1 induced secretion of endogenous SLURP-1 from the intracellular depots (Lyukmanova et al., 2018). Probably, such behavior is also characteristic for healthy epithelial cells of the body, and the paracrine SLURP-1 signaling significantly increases the strength and duration of the antitumor effect. Notably, SLURP-1 and Oncotag do not appear to be toxic to healthy organisms, as evidenced here by toxicity tests and our previous observation of no effects on normal fibroblasts (Bychkov et al., 2021) and keratinocytes (Lyukmanova et al., 2018).

**FIGURE 8:**
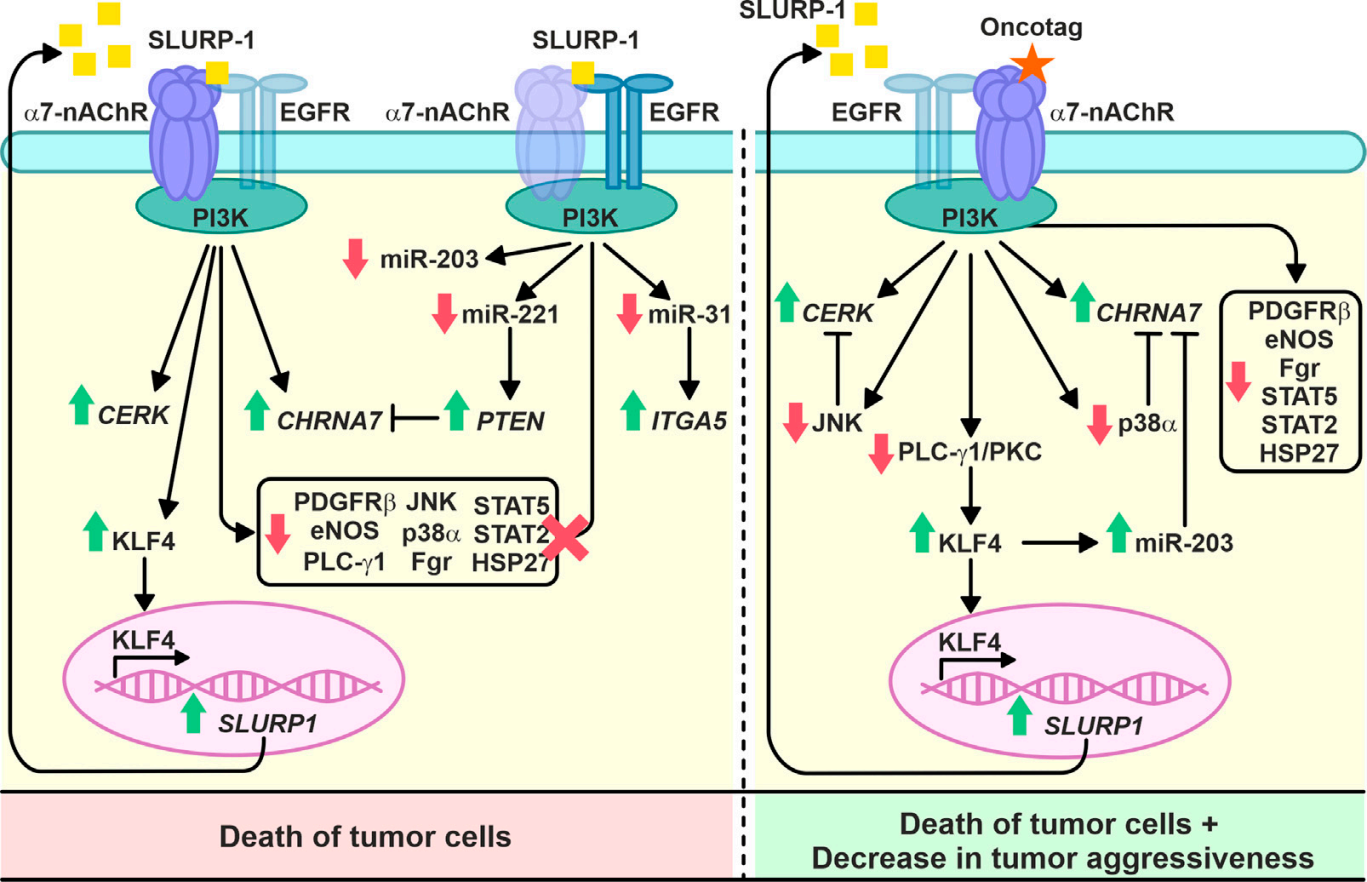
Scheme of the signaling pathways involved in the prolonged SLURP-1 and Oncotag action.

Different effects of SLURP-1 and Oncotag on miR-31, miR-203, miR-221, *ITGA5*, and *PTEN* expression and the mitogenic signaling (Figures 4–6) point on the different signaling pathways triggered by these molecules. Indeed, SLURP-1 targets both α7-nAChR and EGFR, while Oncotag targets only α7-nAChR. That was observed previously in lung cancer A549 cells (Bychkov et al., 2021) and confirmed here for epidermoid carcinoma A431 tumors (Figure 7). Thus, the Oncotag action should lack the signaling events related to EGFR (Figure 8). EGFR and α7-nAChR can either directly interact with each other, or these receptors can be in close proximity in the cell membrane, mutually influencing each other, probably, by interaction with the same intracellular signalling molecules, for example, with PI3K (Chernyavsky et al., 2015; Kharbanda et al., 2020) (Figures 1D, 8). Oncotag inability to extract EGFR from tumors argues in favor of the second possibility.

The antitumor activity of SLURP-1 and Oncotag is probably not limited to action against epidermoid carcinoma. In our previous studies, we have shown that these compounds inhibit proliferation and migration of A549 lung adenocarcinoma cells (Shulepko et al., 2020b; Bychkov et al., 2021). In addition, the efficiency of SLURP-1 has also been demonstrated against various carcinoma and glioma cells *in vitro*, including SKBR3 breast carcinoma, MCF-7 breast carcinoma, HT-29 colorectal adenocarcinoma, and pancreatic ductal adenocarcinoma, and gliomas U251 MG and A172 (Lyukmanova et al., 2014; 2018; Throm et al., 2018; Bychkov et al., 2019; 2021; Shulepko et al., 2020a; 2023). Therefore, we expect that Oncotag and SLURP-1 may be effective in the treatment of various carcinomas and gliomas *in vivo*. Further *in vivo* studies are needed to unlock the full potential of these compounds.

Another important question concerns the long-term effects of SLURP-1 and Oncotag in the body. Although the potential to inhibit tumor growth and metastasis, the long-term use of these compounds may affect the cholinergic system of the organism. Possible long-term side effects of SLURP-1 and Oncotag require further study. Another point of concern is the transfer of the obtained results to the human treatment. All data presented in this work were obtained from the mouse model. Without clinical trials, it is not clear whether SLURP-1 or Oncotag is applicable to the treatment of human tumors. On the other hand, the fact that SLURP-1 is the human protein that appears to control the oncogenic transformation of normal epithelial cells (Arredondo et al., 2007; Kalantari-Dehaghi et al., 2012) gives hope that both SLURP-1 and Oncotag can be safely used in humans.

In conclusion, we studied the antitumor activity and mechanisms of action of the human epithelial protein SLURP-1 and its peptide mimetic Oncotag on epidermoid carcinoma A431 tumor *in vivo*. The tested compounds showed promising results and demonstrated long-lasting antitumor effects. Due to the dual targeting of α7-nAChR and EGFR, SLURP-1 triggers both the pro-oncogenic and anti-oncogenic signaling, while Oncotag activates mainly anti-oncogenic pathways through selective interaction with α7-nAChR (Figure 8). Selective targeting of α7-nAChR with drugs with low systemic toxicity, such as Oncotag, may be a promising strategy for cancer therapy.

## Data Availability

The original contributions presented in the study are included in the article/Supplementary Material, further inquiries can be directed to the corresponding author.
