# Supplementary material for: Selective targeting of α7 nicotinic acetylcholine receptor by synthetic peptide mimicking loop I of human SLURP-1 provides efficient and prolonged therapy of epidermoid carcinoma in vivo
Source: Front Cell Dev Biol. 2023 Oct 3;11:1256716. doi: 10.3389/fcell.2023.1256716 (PMC10580074; doi:10.3389/fcell.2023.1256716)
Supplement: Supplementary file 1 [file DataSheet1.docx]

**Selective targeting of α7-nAChRs by synthetic peptide mimicking loop I of human SLURP-1 provides effective and prolonged therapy of epidermoid carcinoma *in vivo***

O.V. Shlepova^1,2#^, M.A. Shulepko^3,#^, V.O. Shipunova^2,4,#^, M.L. Bychkov^1^, I.D. Kukushkin^2,5^, I.A. Chulina^6^, V.N. Azev^6^, E.I. Shramova^4^, V.A. Kazakov^6^, A.M. Ismailova^6^, Y.A. Palikova^6^, V.A. Palikov^6^, E.A. Kalabina^6^, E.A. Shaykhutdinova^6^, G.A. Slashcheva^6^, E.A. Tukhovskaya^6^, I.A. Dyachenko^6^, A.N. Murashev^6^, S.M. Deyev^4,7,8^, M.P. Kirpichnikov^5,9^, Z.O. Shenkarev^1,2,10^, E.N. Lyukmanova^3,5,9,*^.

^1^NTI Center, Shemyakin-Ovchinnikov Institute of Bioorganic Chemistry, Russian Academy of Sciences, Miklukho-Maklaya str. 16/10, Moscow, Russia

^2^Moscow Institute of Physics and Technology, Institutsky per. 9, Dolgoprudny, Moscow Region, Russian Federation

^3^Faculty of Biology, MSU-BIT Shenzhen University, Shenzhen 518172, China

^4^Immunology Department, Shemyakin-Ovchinnikov Institute of Bioorganic Chemistry, Russian Academy of Sciences, Miklukho-Maklaya str. 16/10, Moscow, Russia

^5^Bioengineering Department, Shemyakin-Ovchinnikov Institute of Bioorganic Chemistry, Russian Academy of Sciences, Miklukho-Maklaya str. 16/10, Moscow, Russia

^6^The Branch of the M.M. Shemyakin Yu.A. Ovchinnikov Institute of Bioorganic Chemistry of the Russian Academy of Sciences, Prospect Nauki 6, Pushchino, Russia

^7^Biomarker" Research Laboratory, Institute of Fundamental Medicine and Biology, Kazan Federal University, 18 Kremlyovskaya St., Kazan 420008, Russia

^8^Sechenov First Moscow State Medical University (Sechenov University), 119991 Moscow, Russia

^9^Interdisciplinary Scientific and Educational School of Moscow University «Molecular Technologies of the Living Systems and Synthetic Biology», Faculty of Biology, Lomonosov Moscow State University, Leninskie Gory, Moscow, Russia

^10^Structural Biology Department, Shemyakin-Ovchinnikov Institute of Bioorganic Chemistry, Russian Academy of Sciences, Miklukho-Maklaya str. 16/10, Moscow, Russia

^#^ Authors with equal contribution

* Corresponding author: lyukmanova_ekaterina@smbu.edu.cn

**Supplementary tables**

**Table S1. Mortality rates for different treatment strategies**

|  | **Control**  **(saline)** | **SLURP-1**  **0.5 mg/kg** | **Oncotag 0.125 mg/kg** | **Oncotag**  **1.25 mg/kg** |
| --- | --- | --- | --- | --- |
| **Number of mice at the beginning of the experiment** | 10 | 10 | 10 | 10 |
| **Number of mice died during the experiment** | 3^1,2,3^ | 1^4^ | 1^5^ | 2^6,7^ |

1-died on 20^th^ day, cause of death is not clear.

2,3- died on 24^th^ day, cause of death is not clear.

4- died on 18^th^ day, necrosis of leg.

5- died on 20^th^ day, cause of death is not clear.

6,7- died on 16^th^ day, cause of death is not clear.

**Table S2. The primers used for qPCR analysis of miRNA expression**

| **miRNA** | **Stem-loop primer** | **Forward primer** | **Reverse primer** |
| --- | --- | --- | --- |
| ***U6 snRNA*** | CGCTTCACGAATTTGCGTGTCA | GCTTCGGCAGCACATATACTAAAAT | CGCTTCACGAATTTGCGTGTCAT |
| ***miR-7*** | CACCGTTCCCCGCCGTCGGTGACAACA | CGCCCTGGAAGACTAGTGAT | CCGTCGGTGACAACAAAAT |
| ***miR-21*** | GTCGTATCCAGTGCAGGGTCCGAGGTATTCGCACTGGATACGACTCAACA | GCCCGCTAGCTTATCAGACTGATG | CAGTGCAGGGTCCGAGGT |
| ***miR-31*** | GTCGTATCCAGTGCAGGGTCCGAGGTATTCGCACTGGATACGACAGCTAT | GCCGCAGGCAAGATGCTGGC |  |
| ***miR-135b*** | GTCGTATCCAGTGCAGGGTCCGAGGTATTCGCACTGGATACGACTCACAT | GCCCGCTATGGCTTTCATTCCT |  |
| ***miR-203*** | GTCGTATCCAGTGCAGGGTCCGAGGTATTCGCACTGGATACGACCTAGTGGTC | GTATCCAGTGCAGGGTCCGA | CGACGGTGAAATGTTTAG |
| ***miR-221*** | CACCGTTCCCCGCCGTCGGTGGAAACC | CGGGCAGCTACATTGTCTG | CGTCGGTGGAAACCAGCA |
| ***miR-451*** | CACGGAACCCCGCCGACCGTGAACTCA | CGCCGAAACCGTTACCAT | GCCGACCGTGAACTCAGTAAT |

**Table S3. The primers used for qPCR analysis of mRNA expression.**

| **mRNA** | **Forward primer** | **Reverse primer** |
| --- | --- | --- |
| ***βACTIN*** | GAGCACAGAGCCTCGCC | CGTACAGGGATAGCACAGCC |
| ***GPDH*** | ATCTTTGTGGTGCCCCATCAGTT | CGCCCTCCATAGCAGGTAGT |
| ***RPL13A*** | TCAAAGCCTTCGCTAGTCTCC | GGCTCTTTTTGCCCGTATGC |
| ***CHRNA7*** | TTTACAGTGGAATGTGTCAGA | TGTGGAATGTGGCGTCAA G |
| ***EGFR*** | GAAATCATACGCGGCAGGAC | TGAGGGAGCGTAATCCCAAG |
| ***PDGFRA*** | TGGAGCTACAGGGAGAGAAAC | GAACGCCGGATGGGAAGTC |
| ***CTNNB1*** | CGTGCACATCAGGATACCCA | GGCTCCGGTACAACCTTCAA |
| ***ITGA2*** | GGTCATCAGGGCACTATCCG | GGCTCCTAAAGGCTCCATCG |
| ***ITGA3*** | GGGCTACCCTATTCCTCCGA | CAGCTCCGAGTCAATGTCCA |
| ***ITGA5*** | GGGCTTCAACTTAGACGCGGA | CCCCAAGGACAGAGGTAGACA |
| ***ITGAV*** | TCCCATCAGTGGTTTGGAGC | AGCTGACGTGATCTACATGG |
| ***CERK*** | TACGTCGAAGCACATGGAGG | TCGTGCAAAGAGTCGAACCA |
| ***PTEN*** | TGTAGTAAGTTGTGCTGAAAGACA | CACCAGTTCGTCCCTTTCCA |
| ***VEGFA*** | AGGAGGAGGGCAGAATCATCA | GGCACACAGGATGGCTTGAA |
| ***SLURP1*** | GGACCATTACCCGCTGCAA | AGGTCTCGGAAGCAGCAGAA |
| ***CDKN1B*** | AATGTTTCAGACGGTTCCCCAAAT | GATGTCCATTCCATGAAGTCAGCG |
| ***KLF4*** | TGCGGCAAAACCTACACAAAG | GTTCATCTGAGCGGGCGAAT |
| ***YWHAZ*** | ACCGTTACTTGGCTGAGGTTG | CAGGCTTTCTCTGGGGAGTTC |
| ***MYC*** | TTCTCTCCGTCCTCGGATTCT | TCCAGACTCTGACCTTTTGCC |
| ***ATF2*** | TACAACAGCCAGCCACATCC | TTAGCTGCTCTTCTCCGACG |
| ***MIF*** | CATCGTAAACACCAACGTGCC | CCGATCTTGCCGATGCTGTG |

**Supplementary figures**


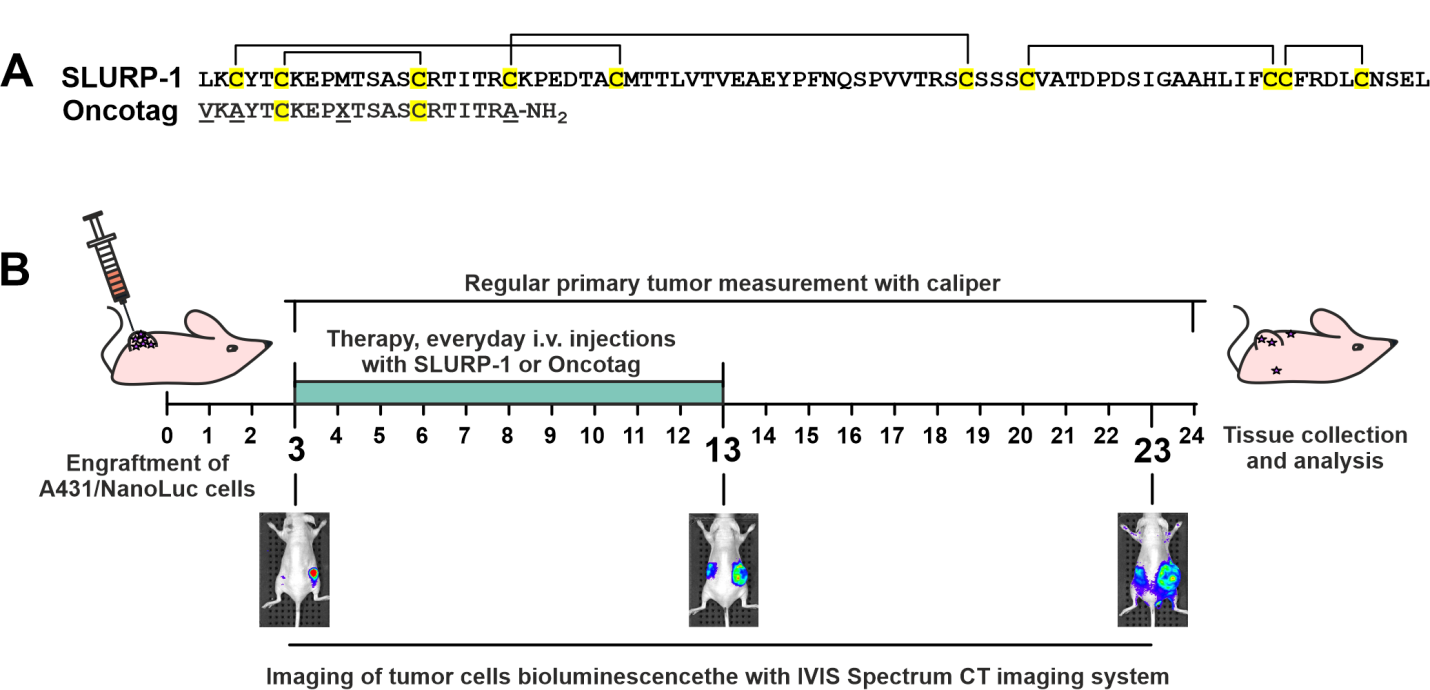


**Fig. S1.** (A) Amino acid sequence alignment of SLURP-1 and the synthetic peptide Oncotag (‘X’ is norleucine, ‘a’ is *C*-terminal amide form). Cysteine residues form a disulfide bridges shown by brackets. Cys residues are shown by yellow. Disulfide bonds are shown by brackets. (B) Scheme of the drugs administration and tumor growth measurements**.**

| The legend tool for spot identification.: | |
| --- | --- |
| 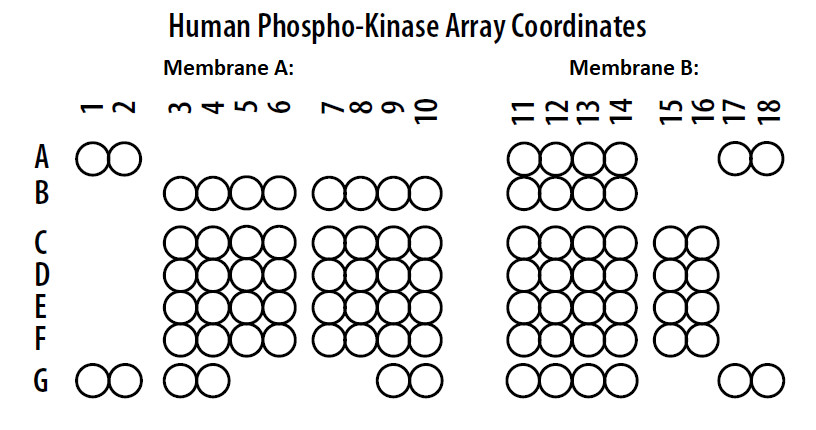 | |
|  | |
| The list of targets with their coordinates (membrane (A,B)-coordinate point 1, point2): | |
| 1. A-A1, A2 Reference Spot 2. A-E3, E4 p38α T180/Y182 3. A-E5, E6 PDGF Rβ Y751 4. A-B3, B4 CREB S133 5. A-B5, B6 EGF R Y1086 6. A-B7, B8 eNOS S1177 7. A-B9, B10 ERK1/2 T202/Y204, T185/Y187 8. A-F3, F4 STAT2 Y689 9. A-E7, E8 PLC-γ1 Y783 10. A-E9, E10 Src Y419 11. A-F5, F6 STAT5a/b Y694/Y699 12. A-F7, F8 WNK1 T60 13. A-C3, C4 Fgr Y412 14. A-F9, F10 Yes Y426 15. A-C5, C6 GSK-3α/β S21/S9 16. A-C7, C8 GSK-3β S9 17. A-C9, C10 HSP27 S78/S82 18. A-G1, G2 Reference Spot 19. A-G3, G4 β-Catenin 20. A-G9, G10 PBS (Negative Control) 21. A-D3, D4 JNK 1/2/3 T183/Y185, T221/Y223 22. A-D5, D6 Lck Y394 | 1. A-D7, D8 Lyn Y397 2. A-D9, D10 MSK1/2 S376/S360 3. B-A11, A12 Akt 1/2/3 T308 4. B-E11, E12 PYK2 Y402 5. B-E13, E14 RSK1/2 S221/S227 6. B-E15, E16 RSK1/2/3 S380/S386/S377 7. B-A13, A14 Akt 1/2/3 S473 8. B-A17, A18 Reference Spot 9. B-B11, B12 Chk-2 T68 10. B-B13, B14 c-Jun S63 11. B-F11, F12 STAT1 Y701 12. B-F13, F14 STAT3 Y705 13. B-F15, F16 STAT3 S727 14. B-C11, C12 p53 S15 15. B-C13, C14 p53 S46 16. B-C15, C16 p53 S392 17. B-G11, G12 STAT6 Y641 18. B-G13, G14 HSP60 19. B-G17, G18 PBS (Negative Control) 20. B-D11, D12 p70 S6 Kinase T389 21. B-D13, D14 p70 S6 Kinase T421/S424 22. B-D15, D16 PRAS40 T246 |

**Fig. S2.** The legend for the spot identification for dot-blot based analysis of phosphorylation of different intracellular factors in A431 cells upon SLURP-1 treatment.


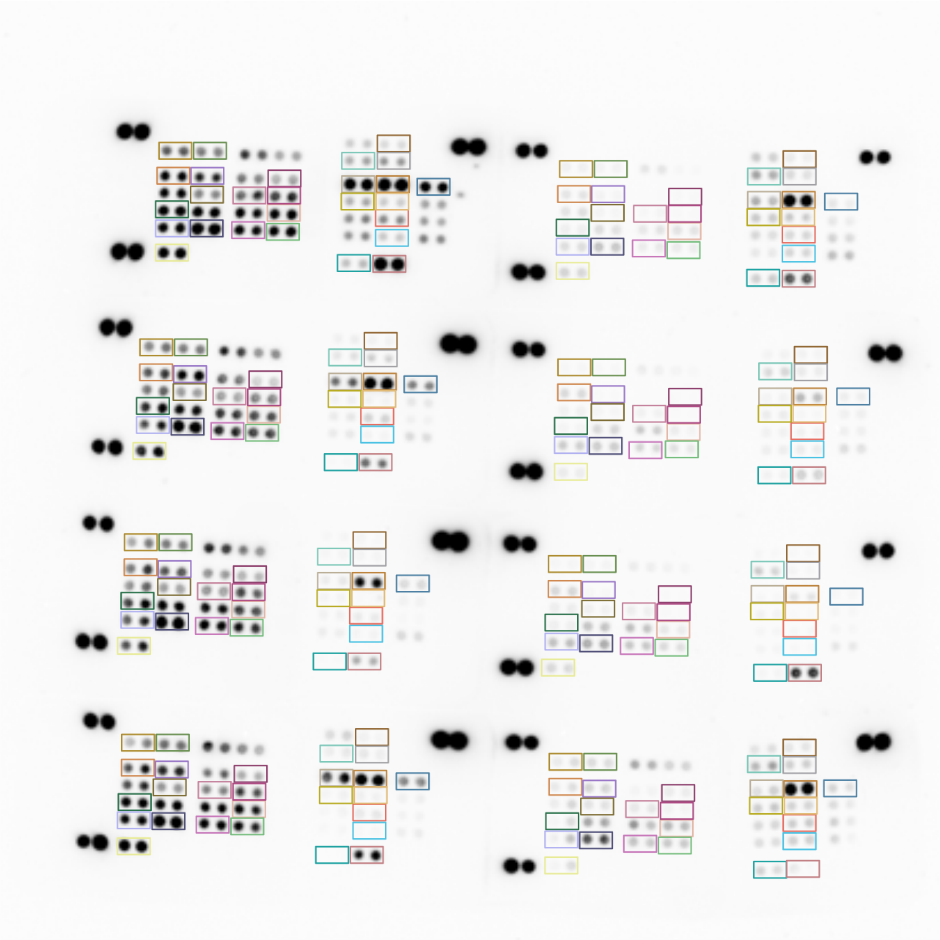


**Figure S3.** The original spots of dot-blot based analysis of phosphorylation of different intracellular factors in A431 cells upon SLURP-1 treatment (n = 4). The membranes with the samples of untreated cells are shown on the left, while the membranes with the samples of cells treated by SLURP-1 are shown on the right. The spots of signal molecules for which significant difference of phosphorylation from the control level was revealed (shown by red asterisks in Fig.2) are marked with color boxes corresponding to the bars in Fig.2.


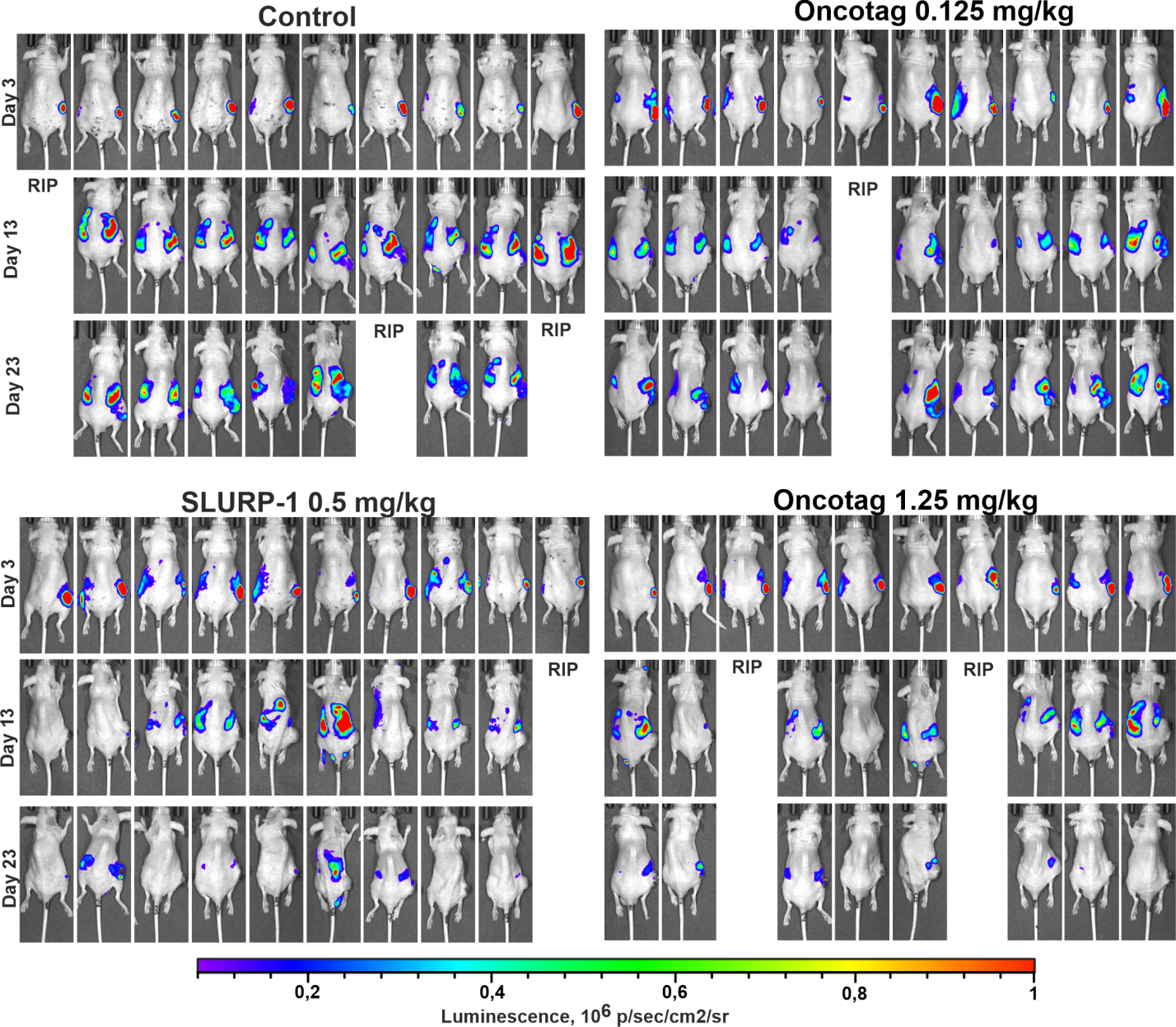


**Fig. S4.** Images of tumor bioluminescence (A431/NanoLuc cells) in mice before treatment (3^rd^ day after tumor engraftment, 1^st^ day of the therapy), after treatment (13^th^ day after tumor engraftment, the next day after end of the 10-day therapy), and before sacrification (23^rd^ day after tumor engraftment). Four groups of animals are used: 1) Control, i.v. injection of saline (n = 7), 2) i.v. injection of 0.5 mg/kg of SLURP-1 (n = 9), 3) i.v. injection of 0.125 mg/kg of Oncotag (n = 9), 4) i.v. injection of 1.25 mg/kg of Oncotag (n = 8). Mice that looked weak and sick were not taken for bioimaging due to for fear of affecting their health. Although they died only a few days later. The day and cause of death are shown in Table S1.

**
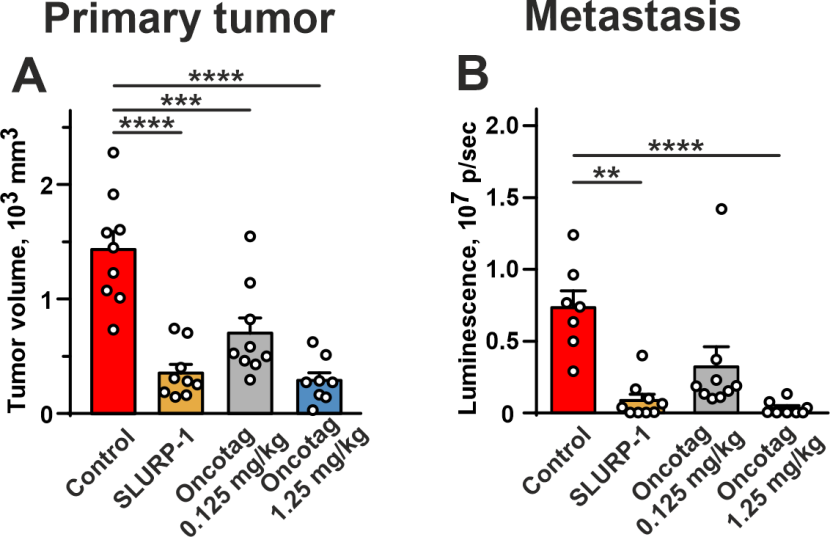
**

**Fig. S5.** Comparison of primary tumor and metastasis volumes for different treatment strategies. (A) Primary tumor volumes measured by a caliper at 24^th^ day after tumor engraftment. Data is presented as mm^3^ ± SEM. *** (p < 0,001), **** (p < 0,0001) indicate the significant difference from the ‘Control’ group (saline) according to the one-way ANOVA test followed by the Dunnett’s post hoc test. (B) Total luminescence measured in the areas away from primary tumor. Data is presented as photons/sec ± SEM. ** (p < 0,01), **** (p < 0,0001) indicate the significant difference from control group (saline) according to the Kruskal-Wallis test followed by the Dunn’s post hoc test.

**
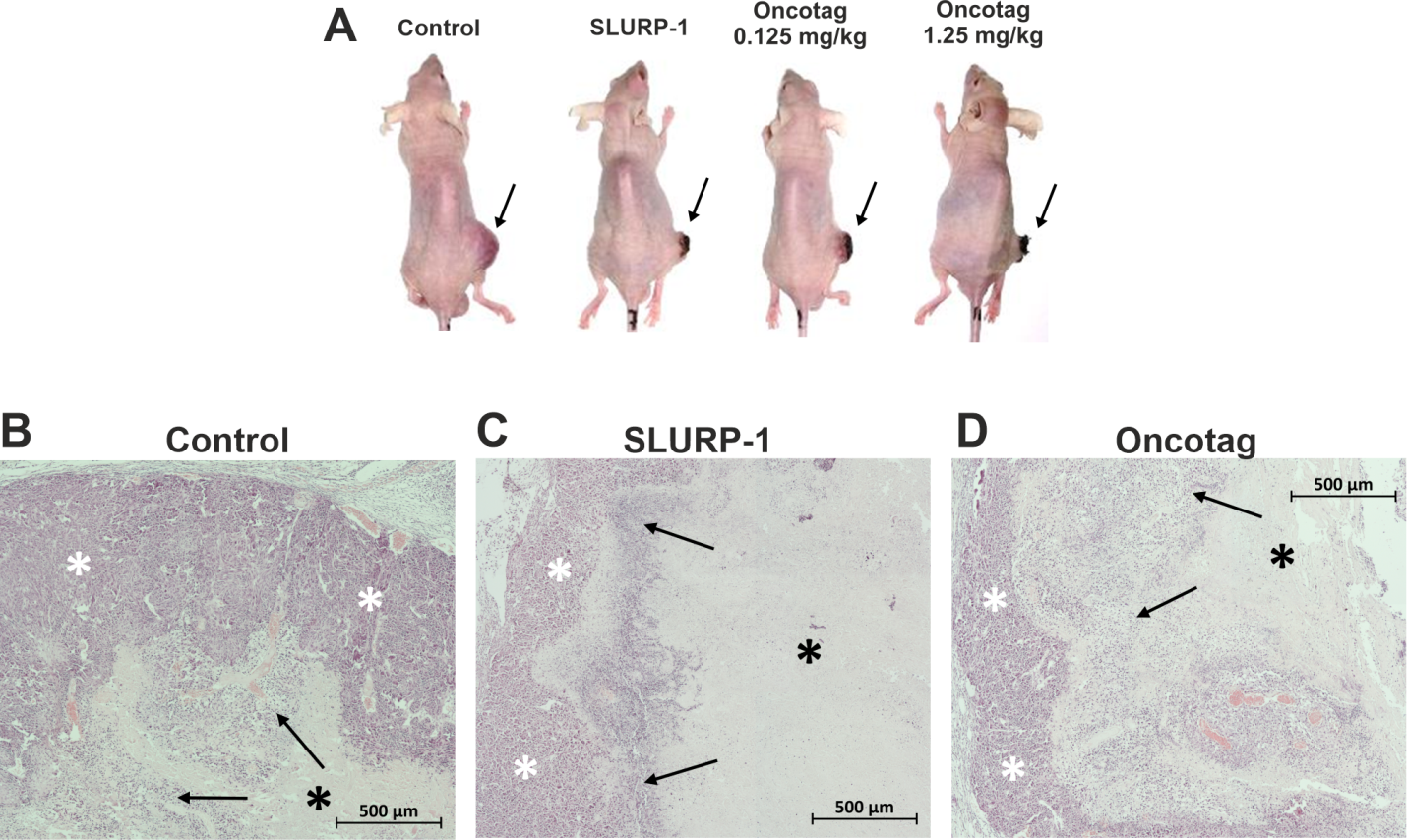
**

**Fig. S6.** The histology analysis of A431/NanoLuc primary tumors. (A) Representative photographs of mice with A431/NanoLuc tumors, primary tumors are indicated by black arrows. Tumors in mice treated with SLURP-1 and Oncotag had necrotic crust in the middle of the primary tumor. (B-D) Representative histology microphotographs of the primary tumors after staining with hematoxylin & eosin. Groups: (B) Control, treatment by saline; (C) treatment by 0.5 mg/kg SLURP-1; (D) treatment by 0.125 mg/kg Oncotag. Tumor cells (white asterisks) are located at the periphery of the tumor, the central part of the tumor is massive areas of necrosis (black asterisk) separated from the layer of poorly differentiated tumor cells by a shaft of mononuclear cells and a few neutrophils (black arrows).


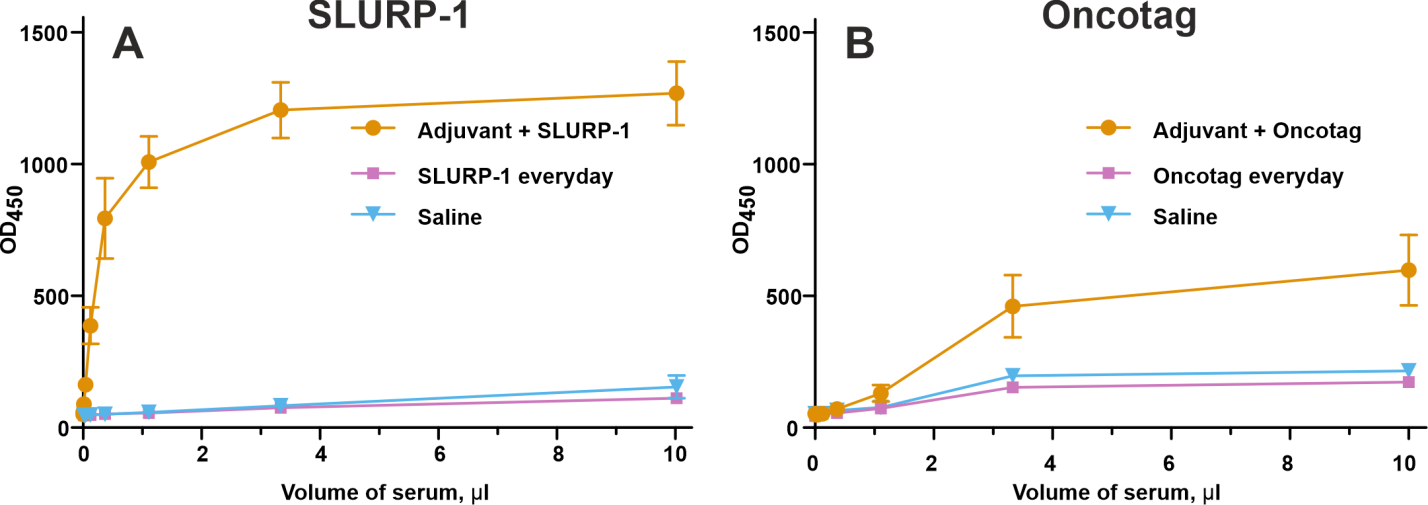


**Fig. S7.** Low immunogenisity of SLURP-1 (A) and Oncotag (B). The presence of antibodies to SLURP-1 in the blood serum of mice was analysed by ELISA. Six group of animals were used (n = 5-10 mice in each group): the 1^st^ and 2^nd^ groups (‘Saline’) received intravenously 10 μl of 0.9% NaCl solution (saline) every day for 5 days; the 3^rd^ and 4^th^ groups received 10 μg of SLURP-1 (0.5 mg/kg) (A) or 2.5 μg of Oncotag (0.125 mg/kg) (B) dissolved in saline every day for 5 days; the 5^th^ and 6^th^ groups received intraperitoneal mixture of 100 µl of complete Freund's adjuvant (BD Biosciences, New Jersey, USA) and 10 µg of SLURP-1 (A) or 2.5 μg of Oncotag (B) on the first day of the study. Blood for ELISA analisys was taken on the 20^th^ day of the study. Data are presented as absorbance at 450 nm. Data are mean ± SEM (n = 5-10).

**
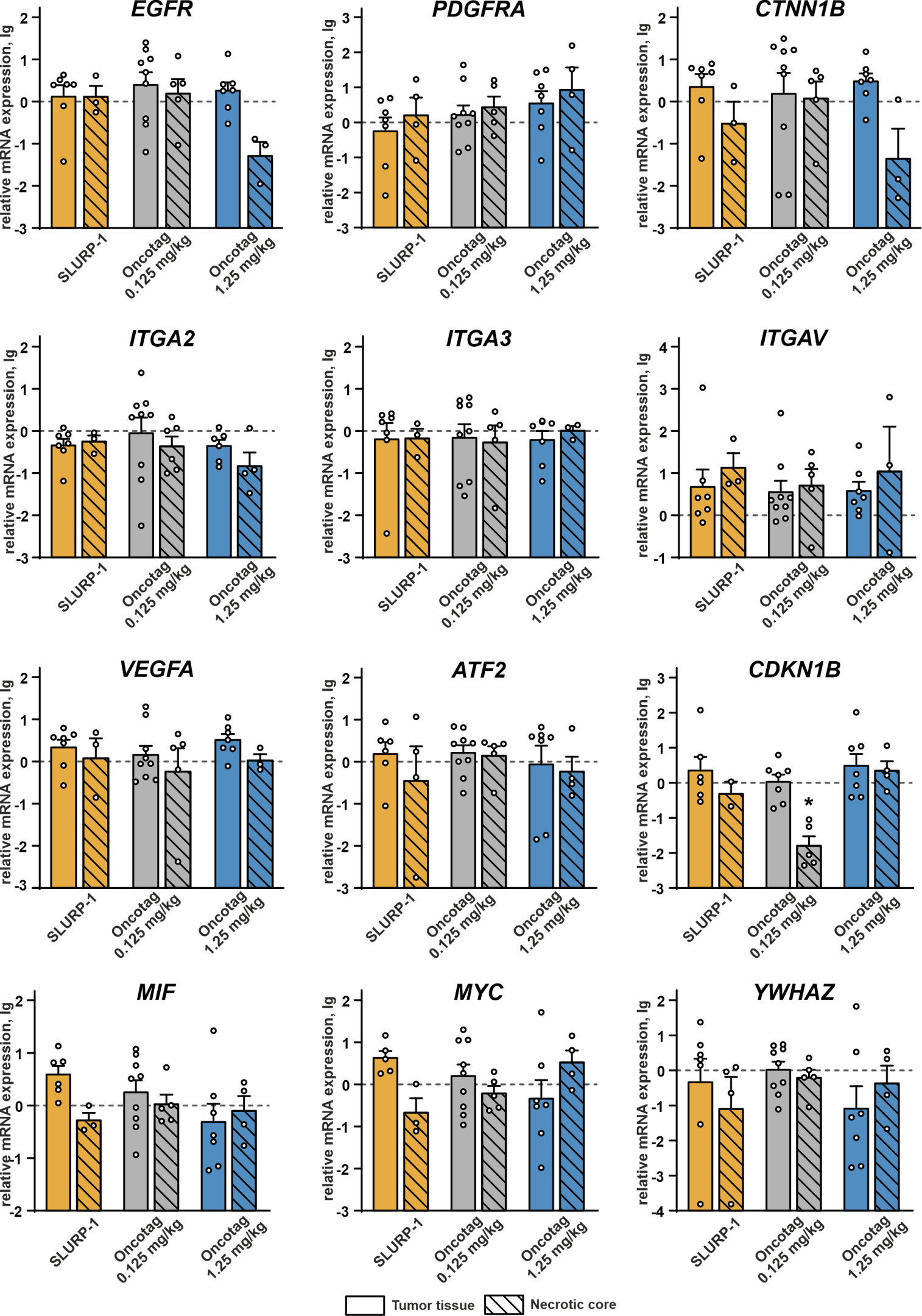
**

**
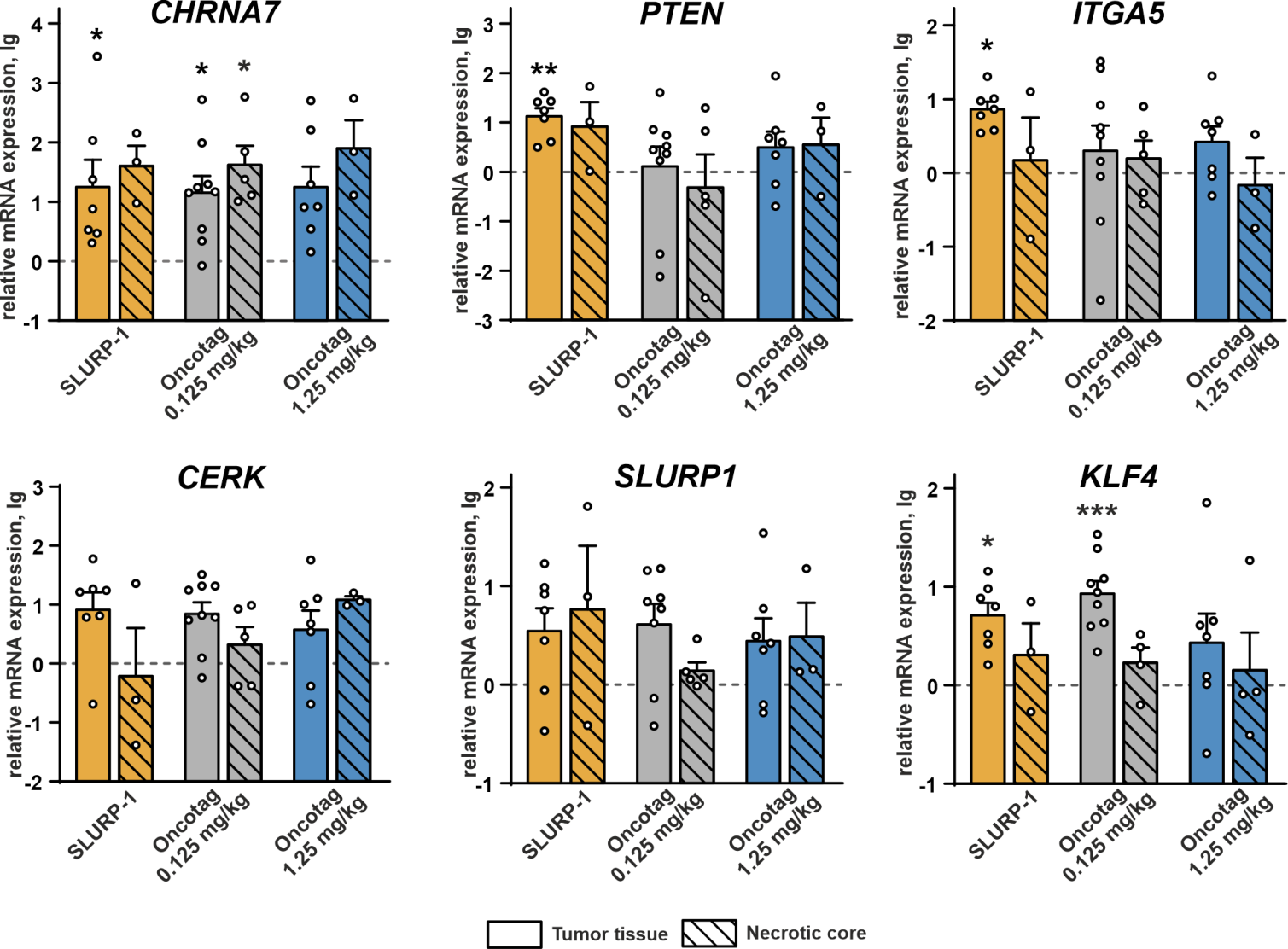
Fig. S8.** Real-time PCR analysis of the mRNA expression of genes in tumors and necrotic tissues after treatment with SLURP-1 (0.5 mg/kg), Oncotag (0.125 mg/kg), and Oncotag (1.25 mg/kg) (n = 3 - 9). The expression of genes coding EGFR (*EGFR*), PDGFRα (*PDGFRA*), β-catenin (*CTNNB1*), integrins α2, α3, α5, and αV (*ITGA2*, *ITGA3*, *ITGA5,* and *ITGAV*, respectively), vascular endothelial growth factor (*VEGFA*), activating transcription factor 2 (*ATF2*), cyclin-dependent kinase inhibitor p27 (*CDKN1B)*, macrophage migration inhibitory factor (*MIF*), MYC protooncogene (*MYC*), tyrosine 3-monooxygenase/tryptophan 5-monooxygenase activation protein ζ (*YWAZ*), α7-nAChR (*CHRNA7*), PTEN (*PTEN*), ceramide kinase (*CERK*), kruppel-like factor 4 (*KLF4*), and SLURP-1 (*SLURP1*) was analyzed. Gene expression was normalized to the expression of *ACTB*, *GAPDH* and *RPL13A* genes of housekeeping proteins. Data are presented as lg of the mRNA expression level normalized to the expression of the same gene in the control group (mice treated with saline, 0, dashed line) ± SEM (n = 3 – 9). * (p < 0.05), ** (p < 0.01), and *** (p < 0.001) indicate significant differences from the control group (0 level) according to a two-tailed one-sample t-test test followed by the Holm-Sidak’s post hoc test.


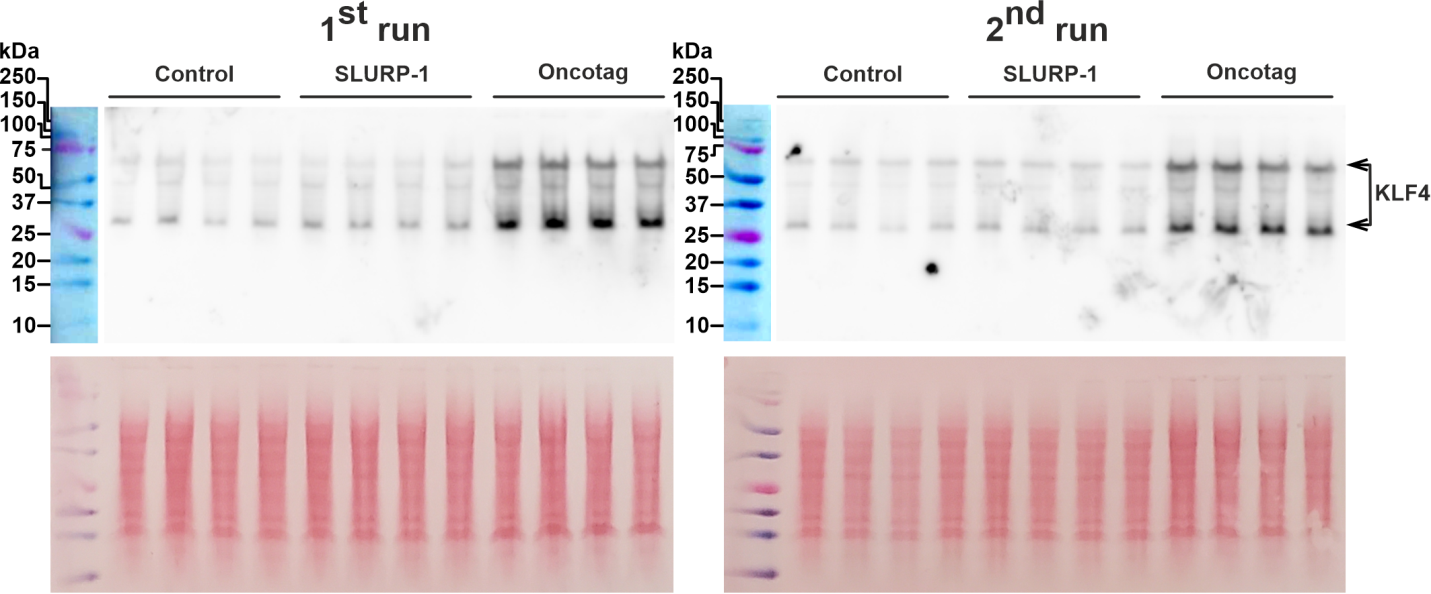


**Fig. S9.** Western blot membranes used for analysis of the KLF4 expression in tumors after saline (control), SLURP-1 (0.5 mg/kg), and Oncotag (0.125 mg/kg) treatment (n = 4 for each group, two different runs). The ECL-imaged membranes used for total protein measurement for each lane are shown below.


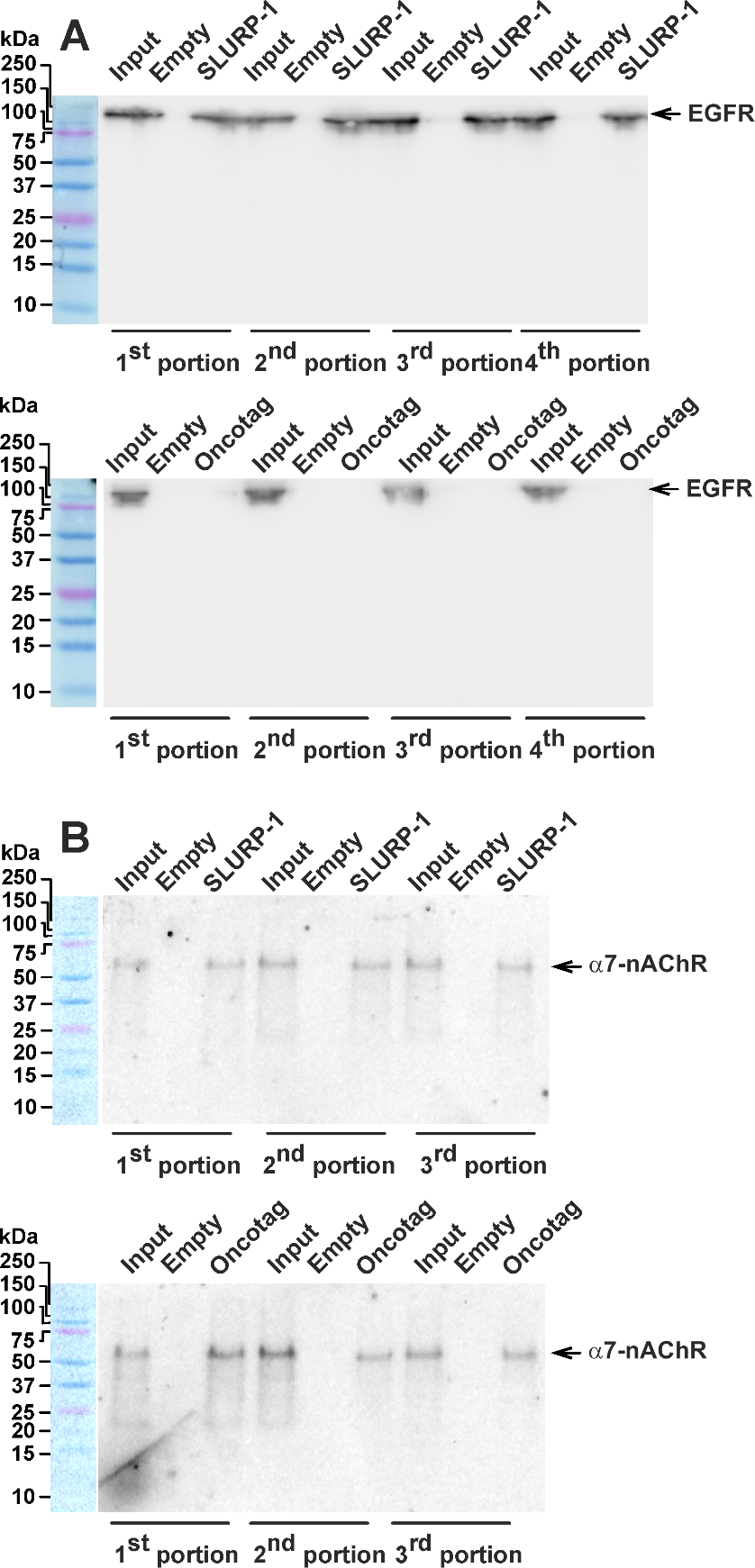


**Fig. S10.** Analysis of SLURP-1 and Oncotag targets in samples of the xenografted A431/NanoLuc tumors of mice. Magnetic beads coupled with SLURP-1 or Oncotag were incubated with a total lysate of the tumor samples from control (saline) group (n = 3-4) and extracted proteins were analyzed by Western blotting using antibodies against EGFR (A) and α7-nAChR (B). For detection of EGFR non-reducing SDS-PAGE was used. Lanes: “Input”– total lysate of the tumor sample used for analysis; “Empty”– proteins extracted from the total lysate by empty magnetic beads without SLURP-1 or Oncotag; “SLURP-1”, “Oncotag” – proteins extracted from the total lysate by magnetic beads coupled with SLURP-1 or Oncotag. Bands corresponding to the EGFR and α7-nAChR are shown by arrows.
